# Targeting GPX2 to disrupt lipid homeostasis and enhance cisplatin sensitivity in diffuse gastric cancer

**DOI:** 10.1038/s41420-025-02771-8

**Published:** 2025-10-27

**Authors:** Yanmei Zhu, Yichun Ma, Wenying Li, Yani Pan, Yue Wang, Qiange Ye, Yinya Pan, Ying Xiang, Ping Jiang, Yi Fang, Lei Xu, Na Liu, Gaifang Liu, Zhangding Wang, Guifang Xu

**Affiliations:** 1 Department of Gastroenterology, Nanjing Drum Tower Hospital, Nanjing Drum Tower Hospital Clinical College of Nanjing University of Chinese Medicine, Nanjing, China; 2 Department of Pathology, Nanjing Drum Tower Hospital, Affiliated Hospital of Medical School, Nanjing University, Nanjing, China; 3 Department of Gastroenterology, Nanjing Drum Tower Hospital, Affiliated Hospital of Medical School, Nanjing University, Nanjing, China; 4 Department of Gastroenterology, Nanjing Drum Tower Hospital, Nanjing Drum Tower Hospital Clinical College of Jiangsu University, Nanjing, China; 5 Department of Gastroenterology, Hebei General Hospital, Shijiazhuang, China; 6 Department of Hepatobiliary Surgery, The First Affiliated Hospital of Anhui Medical University, MOE Innovation Center for Basic Research in Tumor Immunotherapy, Anhui Province Key Laboratory of Tumor Immune Microenvironment and Immunotherapy, Hefei, China; 7 Department of Gastroenterology, The First Affiliated Hospital of Anhui Medical University, Hefei, China

**Keywords:** Gastric cancer

## Abstract

Diffuse gastric cancer (DGC) is characterized by high malignancy and metastasis rate, and poorly understood etiology, culminating in dismal patient outcomes. Here, through comprehensive analysis, we identified that glutathione peroxidase 2 (GPX2) plays a pivotal role in the progression of DGC by regulating lipid metabolism. This study demonstrates that GPX2 is markedly upregulated in DGC tissues, establishing its potential as an independent prognostic indicator. Functionally, GPX2 suppression disrupts lipid droplet formation and lipid homeostasis, leading to increased acylcarnitine levels that impair mitochondrial function. This disruption synergizes with endoplasmic reticulum stress to trigger apoptosis in gastric cancer cells. Notably, inhibiting GPX2 enhances the efficacy of cisplatin by sensitizing cancer cells to apoptosis. These insights identify GPX2 not only as a vital prognostic biomarker but also as a promising therapeutic target for overcoming cisplatin resistance in DGC, offering new avenues for treatment strategies.

## Introduction

Gastric cancer (GC), the third most prevalent and the fifth deadliest gastrointestinal malignancy globally, continues to pose significant challenges in oncology. The Lauren classification systematically delineates GC into the intestinal (IGC), diffuse (DGC), and mixed subtypes, based on morphological, epidemiological, pathological, and genetic characteristics [1]. Notably, DGC, while less common than IGC, has exhibited a rising incidence in recent years [2]. DGC is distinguished by its earlier onset, rapid progression, high metastatic rate, significant familial predisposition, and notably poorer outcomes [3, 4]. In contrast to the better-understood pathogenesis of IGC, the molecular mechanisms underlying DGC remain elusive, highlighting an urgent need for targeted research [5, 6].

The glutathione peroxidase family (GPXs), comprising of antioxidant enzymes, plays critical defense against reactive oxygen species (ROS) and maintaining intracellular redox homeostasis [7]. GPX2 is a member predominantly expressed within the gastrointestinal system, encompassing the epithelial cells of the esophagus, stomach, and intestines [8]. High levels of GPX2 expression have been associated with increased malignancy, invasiveness, and poorer prognosis in various cancers, such as colorectal, pancreatic, lung, and cervical cancers [9–12]. Conversely, GPX2 appears to play a protective and inhibitory role in breast and bladder cancer [13, 14]. Additionally, GPX2 contributes to lenvatinib resistance in hepatocellular carcinoma (HCC) through its influence on oxidative stress and energy metabolism [15]. The activation of the reactive oxygen species (ROS)-mediated KYNU-kyn-AhR signaling pathway, resulting from GPX2 knockdown, has been implicated in the progression and metastasis of gastric adenocarcinoma (GAC) [16]. Despite these insights, the specific role of GPX2 in the progression of DGC remains poorly understood.

Dysregulation of lipid metabolism represents a significant metabolic alteration observed in tumors, essential for maintaining intracellular homeostasis and supporting tumor growth and metastasis [17]. Critical enzymes in the lipid metabolism pathway, such as acetyl coenzyme A carboxylase 1 (ACC1), fatty acid synthase (FASN), ATP citrate lyase (ACLY), and carnitine palmitoyltransferase 1 (CPT1A), are upregulated across a broad spectrum of cancers [18–21]. Lipid droplets (LDs), central lipid storage organelles, extensively interact with mitochondria and serve as the metabolic epicenter for tumor cell proliferation and metastasis [22]. These mitochondria bind to LDs to sequester harmful lipids like diacylglycerol (DAG), cholesterol, ceramide, and polyunsaturated fatty acids, thereby guarding against lipotoxicity and bolstering cellular defenses against apoptosis [23, 24]. Notably, research shows that IGC primarily relies on glucose and glutamine metabolism, whereas the pathogenesis of DGC is predominantly associated with lipid metabolism [25]. This distinction underscores the potential of targeting lipid metabolic pathways as a therapeutic strategy for DGC.

In this study, we conducted a comprehensive search for distinctive DGC markers, leading to the identification of GPX2 as a crucial regulator of lipid metabolism. We discovered that suppressing GPX2 not only hampers the formation of lipid droplets but also leads to elevated levels of acylcarnitine. This accumulation induces mitochondrial dysfunction and triggers apoptosis in GC cells while exacerbating endoplasmic reticulum stress. Moreover, Inhibition of GPX2 significantly increases the susceptibility of GC cells to cisplatin-induced apoptosis. Consequently, our research positions GPX2 as a promising target for anticancer therapy, providing new strategies to overcome cisplatin resistance in DGC. These insights open the door to targeted lipid metabolism therapies in the management of GC, potentially revolutionizing treatment approaches.

## Results

### GPX2 was identified as a biomarker for DGC

**Fig. 1:**
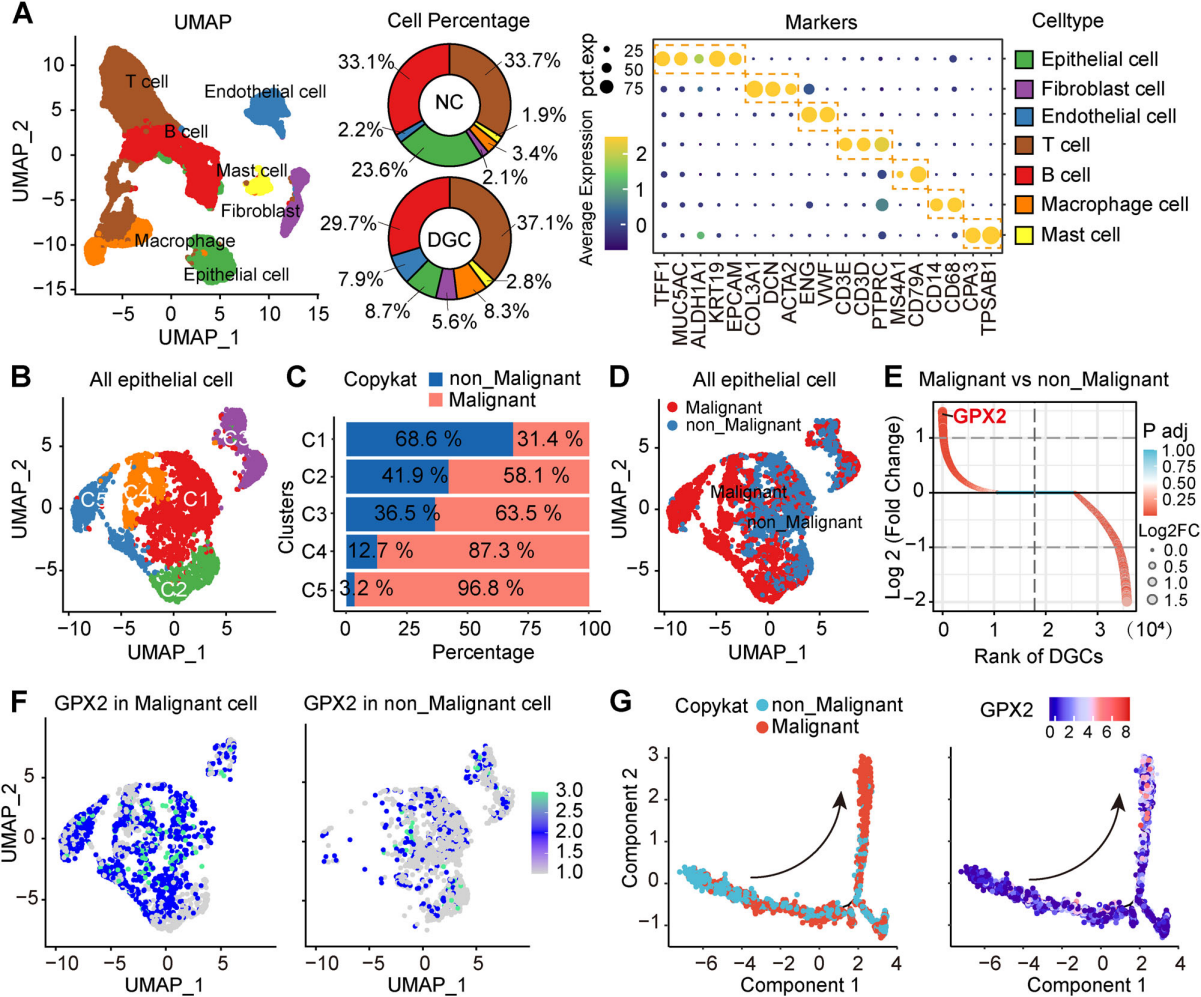
GPX2 was identified as a biomarker for DGC based on single-cell data analysis. **A** Single-cell transcriptomic analysis was conducted on DGC patients (*n* = 10) and normal controls (*n* = 5) utilizing datasets GSE183904 and GSE167297. UMAP analysis identified 39,950 high-quality cells, which were classified into seven distinct cell types. Scaled representation of cell types identified in both normal individuals and DGC patients. Dot plot illustrating representative marker genes for each cell type as delineated by UMAP. **B** UMAP visualization of gastric epithelial cells. **C** A proportional bar chart illustrates the distribution of malignant and non-malignant cells across five epithelial cell clusters. **D** Classification of epithelial cells into malignant and non-malignant groups was achieved using the CopyKAT algorithm, with subsequent visualization via UMAP. **E** Heatmap showcasing differential gene expression (avg_logFC ≥ 1, *p* < 0.05) between malignant and non-malignant gastric epithelial cells. **F** UMAP plot depicting the expression of GPX2 in non-malignant and malignant epithelial cells. **G** Pseudotime analysis tracing the evolution of GPX2 expression from non-malignant to malignant states.

To identify specific gene expression patterns in DGC, we conducted a comprehensive analysis of two single-cell datasets (GSE183904 and GSE167297, Supplementary Fig. 1A and Supplementary Table 1). Following data quality control and integration, a total of 39,950 high-quality cells were retained for further analysis. We employed canonical cell marker genes to classify these cell clusters into seven distinct lineages: epithelial cells, T cells, B cells, fibroblasts, Endothelial cells, mast cells, and macrophages. Cell composition analysis revealed distinct proportions of these seven cell types between normal individuals and patients with DGC (Fig. 1A and Supplementary Fig. 1B, C). Given the origin of GC from gastric mucosal epithelial cells [1], we isolated gastric epithelial cells for in-depth analysis (Fig. 1B and Supplementary Fig. 1D). To evaluate the malignancy of these cells, we applied Copykat [26], an innovative unsupervised algorithm that identifies aneuploid copy number profiles, thereby distinguishing malignant from non-malignant cells (Fig. 1C, D). This approach revealed 518 differentially expressed genes (DEGs) between the two cell states, with significant expression changes (Fig. 1E). Further refinement of these DEGs highlighted GPX2 as the most significantly altered gene, with an expression prevalence of 87.4% in malignant cells compared to only 36.2% in non-malignant cells (Fig. 1F). Additionally, cell trajectory analysis underscored that GPX2 exhibited highly specific expression in subsets of malignant epithelial cells during the transition from non-malignant to malignant states, indicating its critical role in the malignant transformation of DGC (Fig. 1G and Supplementary Fig. 1E, F).

### Elevated GPX2 expression characterizes DGC and correlates with unfavorable prognostic outcomes

**Fig. 2:**
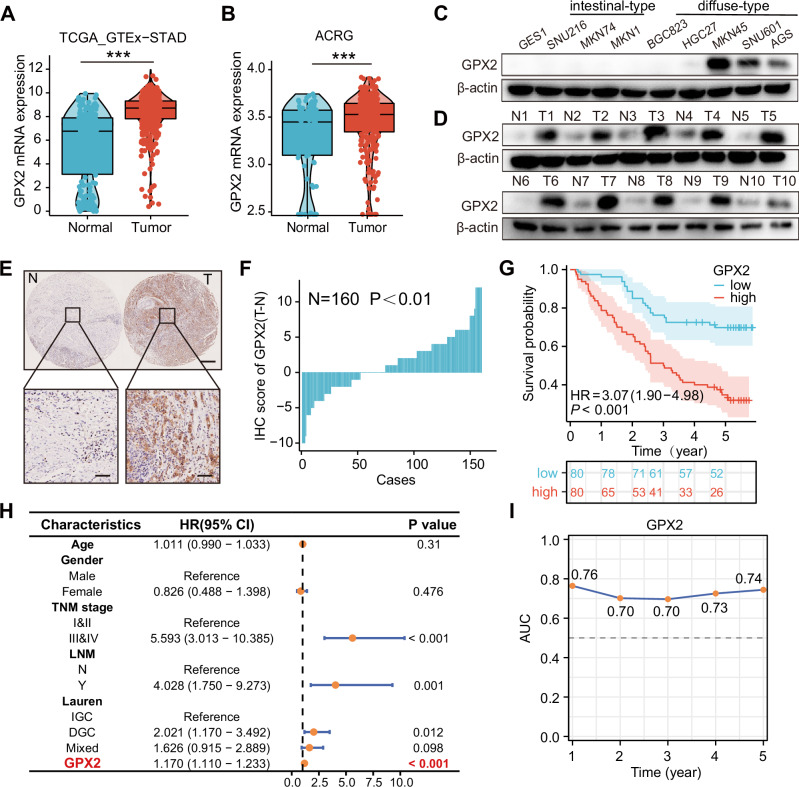
Elevated GPX2 expression in DGC correlates with poor prognostic outcomes. **A** Analysis of GPX2 expression in normal versus tumor tissues using the TCGA-STAD and GTEx databases. **B** GPX2 expression in normal and tumor tissues from the ACRG dataset. **C** Determination of GPX2 protein levels in gastric cancer cell lines via Western blot analysis. **D** Measurement of GPX2 protein levels in tumor tissues and adjacent non-tumor tissues from DGC patients using Western blot. **E** Immunohistochemical staining of GPX2 in tissue microarray composed of cancerous and adjacent non-cancerous tissues from 160 gastric cancer patients. Scale bars = 50 μm or 200 μm. **F** Distribution of the difference in immunohistochemistry (IHC) scores (ΔIRS = IRST–IRSN) for GPX2 in tissue microarrays. **G** Kaplan–Meier survival curves for gastric cancer patients with high and low GPX2 expression. **H** Univariate Cox regression analysis assessing the prognostic significance of GPX2 expression. **I** Time-dependent AUC curve of GPX2.

To further confirm GPX2 expression in DGC, we analyzed its levels in the gastric adenocarcinoma (STAD) cohort from The Cancer Genome Atlas (TCGA) and the Asian Cancer Research Group (ACRG), and the results revealed heightened increased GPX2 expression in GC (Fig. 2A, B). Notably, compared to non-malignant GES1 and IGC cell lines (MKN74, SNU216, and MKN1) [27, 28], DGC cell lines (AGS, SNU601, and MKN45) [28–30] exhibited significantly higher GPX2 expression (Fig. 2C). Similarly, GPX2 protein levels were markedly increased in cancerous tissues compared to normal tissues (Fig. 2D).

To assess the correlation between GPX2 expression and prognosis, immunohistochemical staining was conducted on tissue microarray from 160 GC patients (Supplementary Table 2), and survival analysis revealed a poorer prognosis in patients with high GPX2 expression (Fig. 2E–G). Subsequent stratification of GC patients into high and low GPX2 expression groups showed an association between GPX2 expression levels and various clinicopathological features. Univariate Cox analysis identified GPX2 protein expression, TNM stage, lymph node metastasis, and Lauren classification as prognostic risk factors for GC (Fig. 2H). Evaluation of the predictive ability of GPX2 expression for survival in GC via ROC curve analysis yielded the time-dependent area under the curve (AUC) was greater than 0.7 (Fig. 2I) [31]. These findings indicate that GPX2 is highly expressed in DGC and is associated with poor prognosis, suggesting its potential as an independent prognostic risk factor for GC.

### GPX2 promotes lipid droplet formation and regulates lipid synthesis

To investigate the function of GPX2 in the DGC, we established stable knockdown and GPX2 overexpression cell lines, and the efficiency was confirmed at both RNA and protein levels (Supplementary Figs. 2A–D and 3A–D). CCK-8 and colony formation assays demonstrated that GPX2 silencing inhibited GC cell proliferation (Supplementary Fig. 2E–H), whereas GPX2 overexpression enhanced cell proliferation (Supplementary Fig. 3E–H). Moreover, GPX2 overexpression significantly promoted GC cell migration and invasion capabilities (Supplementary Figs. 2I, J and 3I, J). Collectively, these findings suggest that GPX2 may contribute to the tumorigenesis and malignant progression.

**Fig. 3:**
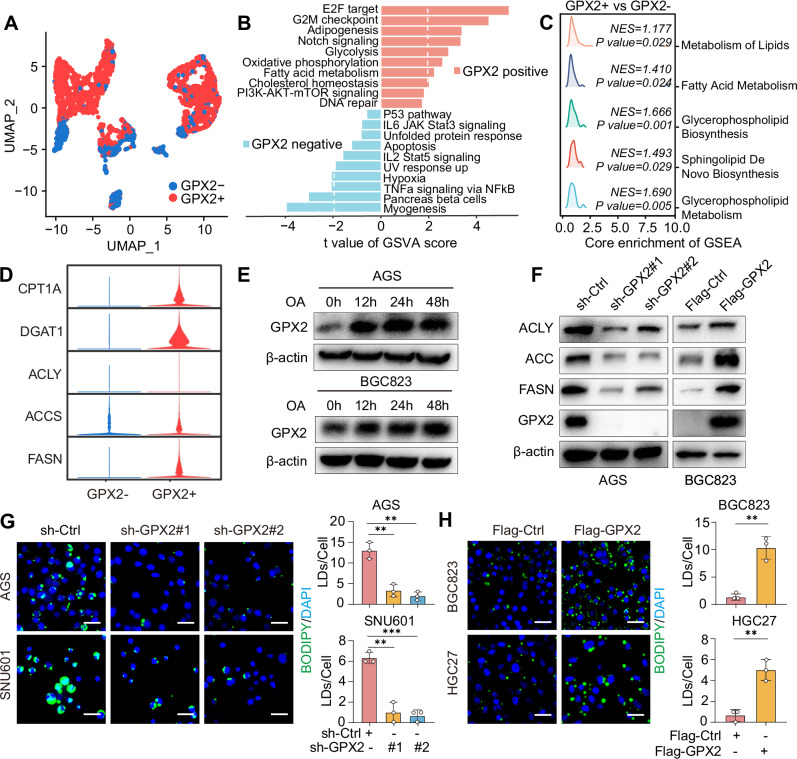
Silencing GPX2 expression inhibits lipid droplet formation and modulates lipid biosynthesis. **A** UMAP plot of GPX2-positive and GPX2-negative subsets of malignant gastric epithelial cells. **B** Results of gene set variation analysis (GSVA) for GPX2-positive and GPX2-negative cells. **C** The GSEA plot of differentially expressed genes between GPX2-positive and GPX2-negative cells. **D** Violin plot illustrating the expression of lipid metabolism markers in GPX2-positive and GPX2-negative subsets. **E** Time-course analysis of GPX2 protein levels in AGS and BGC823 cells stimulated with 200 μM oleic acid for 0, 12, 24, and 48 h. **F** Western blot analysis of lipid metabolism-related gene expression in GPX2-knockdown AGS and GPX2-overexpressed BGC823 cells. **G** BODIPY 493/503 fluorescence staining of GPX2-knockdown AGS and SNU601 cells, stimulated by 200 μM oleic acid for 24 h, illustrating the impact on lipid droplet production. Scale bars = 50 μm. **H** BODIPY 493/503 fluorescent dye was used to visualize lipid droplets in the GPX2-overexpressed BGC823 and HGC27 cells. Scale: 50 μm.The data are represented as the mean ± SD of three independent experiments. * *p* < 0.05; ** *p* < 0.01; *** *p* < 0.001, ns no significance.

To further elucidate how GPX2 promotes DGC malignancy, we stratified malignant epithelial cells into GPX2-positive and GPX2-negative subclusters (Fig. 3A). Gene Set Variation Analysis (GSVA) of these subsets unveiled notable enrichment of oxidative phosphorylation, adipogenesis, and fatty acid metabolism pathways in GPX2-positive cells (Fig. 3B). GSEA enrichment analysis revealed differences in various lipid metabolic pathways between GPX2-positive and GPX2-negative cells (Fig. 3C). Moreover, the expression levels of pivotal lipid synthesis regulators, including ACCs, ACLY, and DGAT1, were markedly elevated in GPX2-positive cells compared to GPX2-negative cells, suggesting a potential regulatory role of GPX2 in lipid metabolism (Fig. 3D). To investigate this hypothesis, we subjected GC cells to Oleic Acid (OA) treatment in a time-dependent manner and observed a progressive augmentation in GPX2 protein expression, implying a probable function of GPX2 as an OA receptor in GC cells (Fig. 3E). Subsequent Western blot analysis validated that GPX2 regulates the expression of lipid synthesis-related proteins in GC cells (Fig. 3F and Supplementary Fig. 4C). Additionally, we explored the influence of GPX2 on lipid droplet biogenesis using Oil Red O (Supplementary Fig. 4A, B) and BODIPY493/503 staining (Fig. 3G, H). Our results demonstrated that GPX2 knockdown significantly diminished the number of lipid droplets in GC cells, whereas GPX2 overexpression markedly augmented lipid droplet formation. Additionally, we performed GPX2 rescue experiments by reintroducing GPX2 via lentiviral overexpression in GPX2-knockdown gastric cancer cells, and the results demonstrated that GPX2 reconstitution significantly reversed the reduced lipid droplet formation induced by GPX2 knockdown (Supplementary Fig. 4D–F). These findings collectively suggest that GPX2 intricately regulates lipid synthesis and lipid droplet biogenesis in GC cells.

### Mitochondrial dysfunction and endoplasmic reticulum stress induced by GPX2 inhibition trigger DGC cell apoptosis

**Fig. 4:**
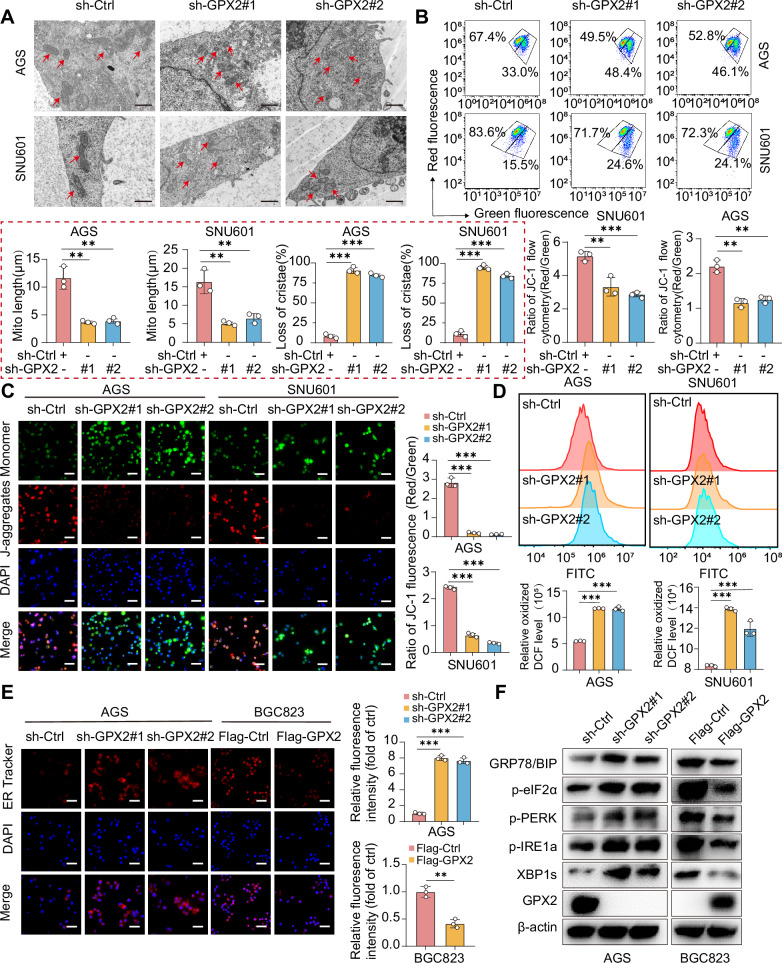
Silencing GPX2 expression induces mitochondrial dysfunction and exacerbates endoplasmic reticulum stress. **A** Transmission electron microscopy revealed the mitochondrial structure of GPX2-knockdown AGS and SNU601 cells. Red arrows indicate mitochondria. Scale: 1 μm. **B** Flow cytometry was utilized to assess the mitochondrial membrane potential (JC-1) in AGS and SNU601 cells with GPX2 knockdown. **C** JC-1 fluorescence staining depicted the mitochondrial membrane potential levels in GPX2-down AGS and SNU601 cells. Scale bars = 50 μm. **D** The levels of reactive oxygen species (ROS) in AGS and SNU601 cells with GPX2 knockdown were measured by flow cytometry. **E** ER-Tracker fluorescence staining illustrated the endoplasmic reticulum structure in GPX2-knockdown AGS and GPX2-overexpressed BGC823 cells. Scale bars = 50 μm. **F** Western blot analysis was employed to evaluate the levels of endoplasmic reticulum (ER) stress-related proteins. The data are represented as the mean ± SD of three independent experiments. * *p* < 0.05; ** *p* < 0.01; *** *p* < 0.001, ns no significance.

Lipid droplets and imbalance of cellular lipid homeostasis often accompany mitochondrial dysfunction and alterations in mitochondrial morphology [24]. To identify the role of GPX2 in maintaining mitochondrial homeostasis, we conducted transmission electron microscopy (TEM) analysis of DGC cellular morphology. Results revealed that GPX2 inhibition severely compromised mitochondrial structure, leading to fragmented, rounded mitochondria lacking cristae compared to the elongated tubular shape observed in control cells (Fig. 4A). Flow cytometric analysis further demonstrated a reduction in mitochondrial membrane potential post-GPX2 knockdown (Fig. 4B), corroborated by JC-1 staining which showed a decrease in red fluorescent polymers within the mitochondrial matrix and an increase in green fluorescent monomers (Fig. 4C), indicative of diminished mitochondrial membrane potential. Additionally, GPX2 inhibition resulted in oxidative stress, evidenced by a significant increase in reactive oxygen species (ROS) production (Fig. 4D), further exacerbating mitochondrial dysfunction. Restoration of GPX2 expression in knockdown GC cells rescued the GPX2 knockdown-induced mitochondrial dysfunction, as evidenced by normalized ROS production (Supplementary Fig. 5A, B) and recovered mitochondrial membrane potential (Supplementary Fig. 5C, D), establishing a key role of GPX2 in mitochondrial homeostasis. Interestingly, unfolded protein response (UPR) is a cytoprotective signaling pathway triggered by mitochondrial dysfunction, prompting us to assess the involvement of GPX2 in this process [32]. We utilized ER-Tracker staining to monitor the endoplasmic reticulum (ER) in GPX2-knockdown cells, revealing ER expansion indicative of ER stress, while GPX2 overexpression appeared to stabilize the ER (Fig. 4E and Supplementary Fig. 6A). Furthermore, GPX2 knockdown activated UPR sensors, including PERK, IRE1α, and eIF2α, and upregulated various UPR target genes, as evidenced by molecular analyses. Conversely, GPX2 overexpression exerted opposite effects, indicating a regulatory role of GPX2 in modulating the UPR pathway (Fig. 4F and Supplementary Fig. 6B). Immunofluorescence colocalization of lipid droplet marker PLIN2 [33, 34] and ER stress marker GRP78 revealed significant overlap (Supplementary Fig. 6C), underscoring the mechanistic link between lipid dysregulation and ER stress.

**Fig. 5:**
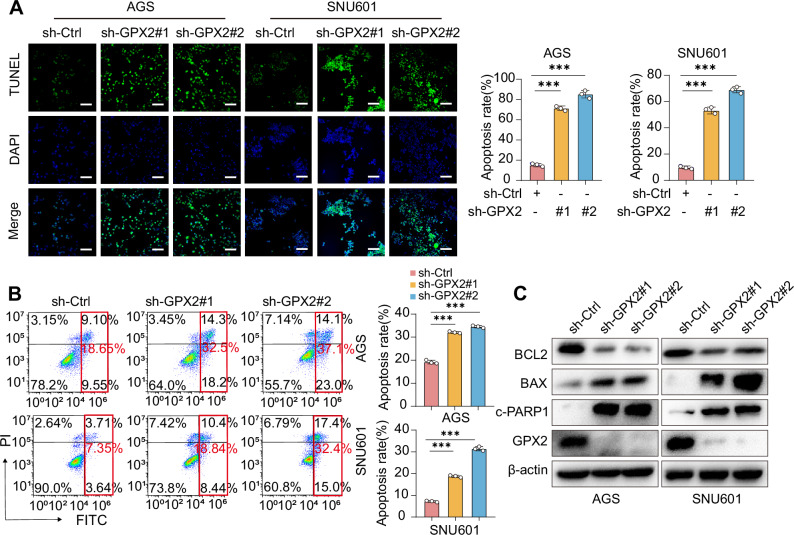
Knockdown of GPX2 enhances apoptosis in gastric cancer cells. **A** TUNEL assay was employed to assess the apoptosis levels in AGS and SNU601 cells following GPX2 knockdown. Scale bars = 100 μm. **B** Flow cytometry was utilized to examine the impact of GPX2 knockdown on apoptosis in AGS and SNU601 cells. **C** Western blot analysis was conducted to evaluate the expression of apoptosis-related proteins in GPX2-knockdown AGS and SNU601 cells. The data are represented as the mean ± SD of three independent experiments. * *p* < 0.05; ** *p* < 0.01; *** *p* < 0.001, ns no significance.

Indeed, both exaggerated mitochondrial damage and endoplasmic reticulum stress can precipitate apoptosis [35–37]. To investigate the impact of GPX2 on apoptosis, we utilized the TUNEL assay and flow cytometry (Fig. 5A, B), and the results revealed a significant increase in the number of apoptotic GC cells following GPX2 knockdown, accompanied by an upregulation of pro-apoptotic gene proteins BAX and cleaved PARP1, and downregulation of the anti-apoptotic gene BCL2 (Fig. 5C). Critically, GPX2 reconstitution attenuated apoptosis (Supplementary Fig. 7A–C), confirming its anti-apoptotic function. To mechanistically dissect the GPX2-ER stress-apoptosis axis, we treated GPX2-knockdown GC cells with the ER stress inhibitor 4-PBA [38]. 4-PBA reversed both ER stress exacerbation and apoptotic induction (Supplementary Figs. 8A–C and 9A–C) triggered by GPX2 loss. These results demonstrate that GPX2 silencing drives apoptosis in DGC through coordinated mitochondrial dysfunction and ER stress potentiation.

### GPX2 inhibition disrupts lipid homeostasis and increases cellular acylcarnitine levels

**Fig. 6:**
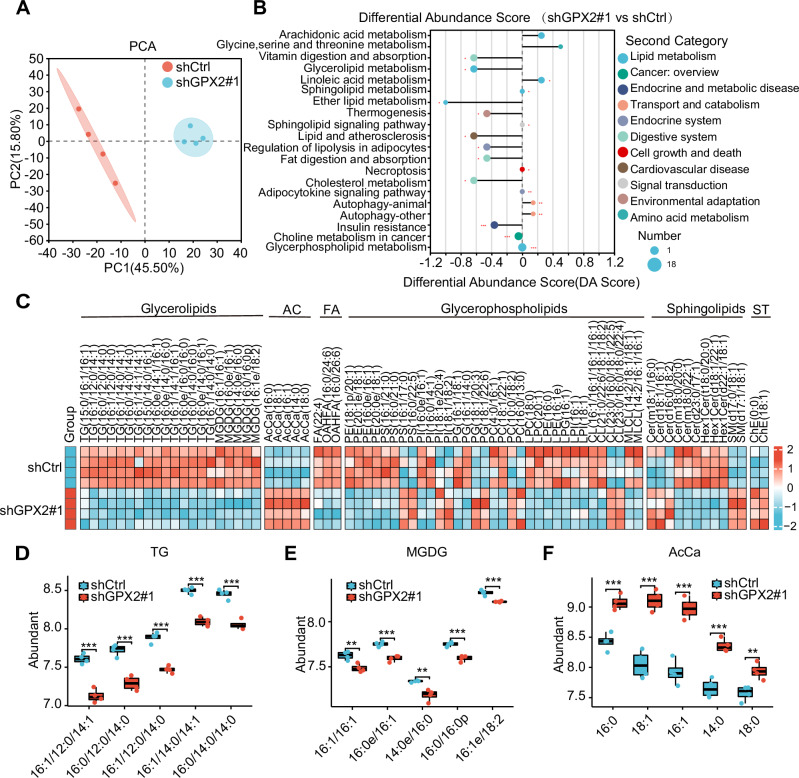
GPX2 modulates lipid homeostasis, with GPX2 silencing notably elevating acylcarnitine levels in GC cells. **A** Principal component analysis (PCA) of AGS cells in the control group and GPX2 knockdown group. **B** Differential abundance scores of lipid metabolites. **C** Heatmap representing the lipid profiles in control and GPX2-knockdown AGS cells. **D** Abundance values of triglycerides, **E** digalactosylglycerides, and **F** acylcarnitines in control and GPX2-knockdown AGS cells. The data are represented as the mean ± SD of three independent experiments. * *p* < 0.05; ** *p* < 0.01; *** *p* < 0.001, ns no significance.

To elucidate the mechanisms underlying mitochondrial dysfunction, endoplasmic reticulum (ER) stress, and cellular apoptosis associated with GPX2 inhibition, we performed lipidomic sequencing on DGC cells. Principal component analysis (PCA) was employed to differentiate the lipid profiles between control (shCtrl) and GPX2-knockdown (shGPX2) groups, revealing distinct clustering (Fig. 6A). We observed markedly reduced lipid metabolic pathway abundance scores in the GPX2-knockdown cells compared to controls (Fig. 6B), indicating a disruption in lipid homeostasis. Additionally, Significant alterations were noted in the levels of major lipid classes, including glycerides, fatty acids, glycerolipids, and sphingomyelins (Fig. 6C), which underscore the integral role of GPX2 in lipid metabolism regulation within GC cells. Notably, the levels of triglycerides and diglycerides were significantly reduced in the GPX2-knockdown cells (Fig. 6D, E). In contrast, acylcarnitines were significantly elevated (Fig. 6F), supporting the hypothesis that GPX2 inhibition leads to impaired lipid synthesis and biosynthesis of lipid droplets. This impairment results in the excessive release of free fatty acids and their abnormal diversion to acylcarnitine production.

### GPX2 knockdown results in elevated levels of acylcarnitine, which is associated with mitochondrial dysfunction and apoptosis

**Fig. 7:**
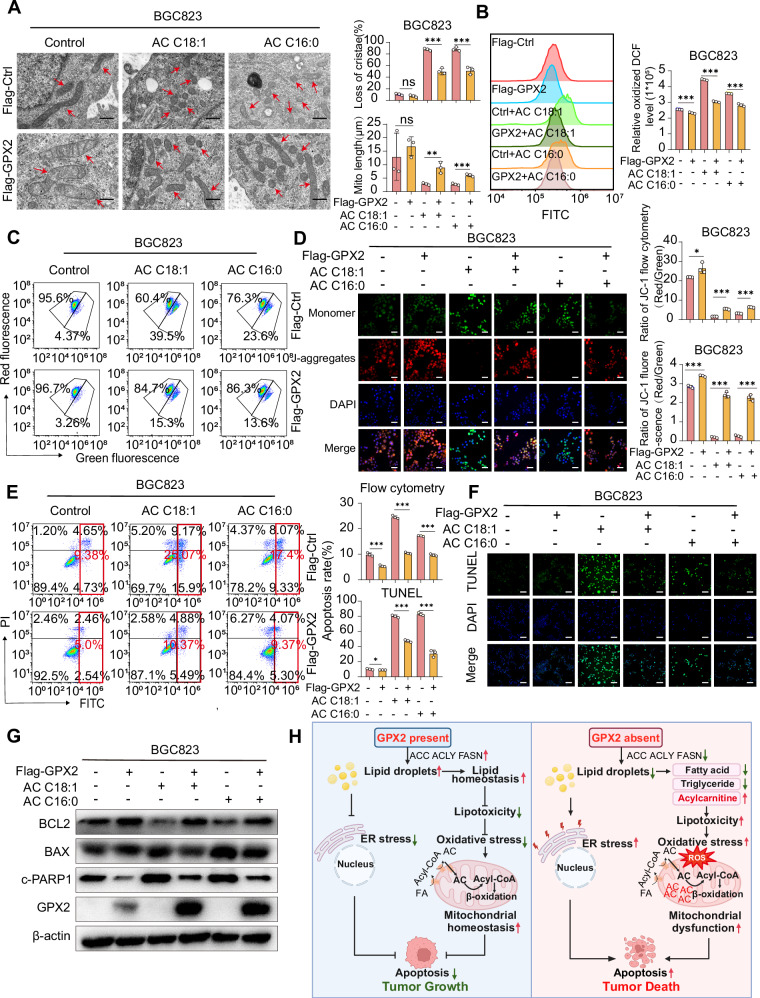
GPX2 knockdown results in elevated levels of acylcarnitine, which is associated with mitochondrial dysfunction and apoptosis. BGC823 transfected with control and GPX2-overexpressing viruses, were respectively treated with 30 μM C18:1 oleoyl L-carnitine and 20 μM C16:0 palmitoyl L-carnitine for 24 h. **A** Transmission electron microscopy revealed the structure of cell mitochondria. Red arrows indicate mitochondria. Scale bars = 500 nm. **B** Flow cytometry was utilized to detect the levels of reactive oxygen species (ROS). **C** Changes in mitochondrial membrane potential were assessed by flow cytometry. **D** JC-1 fluorescence staining indicated the levels of mitochondrial membrane potential. Scale bars = 50 μm. Following treatment with 30 μM C18:1 oleoyl L-carnitine and 20 μM C16:0 palmitoyl L-carnitine for 48 h: **E** Apoptotic rates were quantified using flow cytometry. **F** Apoptosis levels were measured by the TUNEL assay. Scale bars = 100 μm. **G** Quantitative analysis of apoptosis-related protein levels were conducted using Western blot. **H** Schematic representation of the GPX2 regulatory mechanism (Graphic created with BioRender.com). The data are represented as the mean ± SD of three independent experiments. * *p* < 0.05; ** *p* < 0.01; *** *p* < 0.001, ns no significance.

Acylcarnitine, a fatty acid conjugate formed in the outer membrane of mitochondria, is essential for the β-oxidation of fatty acids into mitochondria [39]. When elevated, is hypothesized to lead to mitochondrial dysfunction and subsequently induce apoptosis in GC cells. To test this hypothesis, we exposed HGC27 and BGC823 GC cell lines, exhibiting normal and stable overexpression of GPX2, respectively, to C18:1 AC (oleoyl L-carnitine) and C16:0 AC (palmityl L-carnitine). TEM investigations revealed that acylcarnitine supplementation induced structural disruptions in mitochondria and loss of cristae (Fig. 7A and Supplementary Fig. 10A). This was accompanied by an increase in cellular reactive oxygen species (ROS) levels (Fig. 7B and Supplementary Fig. 10B) and a decrease in mitochondrial membrane potential (Fig. 7C, D and Supplementary Fig. 10C, D). Interestingly, cells with GPX2 overexpression exhibited resistance to these deleterious effects, suggesting that GPX2 plays a protective role against acylcarnitine-induced mitochondrial damage. Further experiments to assess apoptosis via TUNEL assay and flow cytometry confirmed a significant increase in apoptotic cell death following acylcarnitine treatment (Fig. 7E, F and Supplementary Fig. 10E, F). This effect was notably mitigated in cells overexpressing GPX2. Additionally, treatment with acylcarnitine resulted in increased expression of pro-apoptotic genes such as BAX and C-PARP1, while decreasing the expression of the anti-apoptotic gene BCL2. However, GPX2 overexpression effectively reversed the apoptotic effects induced by acylcarnitine (Fig. 7G and Supplementary Fig. 10G). To further determine whether elevated acylcarnitine levels serve as the primary driver of mitochondrial dysfunction and apoptosis, we targeted its biosynthesis using mildronate [40] in GPX2-knockdown cells. Our results demonstrated that pharmacological inhibition of acylcarnitine biosynthesis significantly attenuated both mitochondrial damage (Supplementary Fig. 11A–D) and cell apoptosis (Supplementary Fig. 11E–G) in GPX2-knockdown cells. These above results showed that GPX2 knockdown elevated acylcarnitine levels, resulting in mitochondrial dysfunction and, consequently, promoting apoptosis in GC cells.

### Elevated expression of GPX2 can counteract apoptosis induced by cisplatin and diminish drug sensitivity

**Fig. 8:**
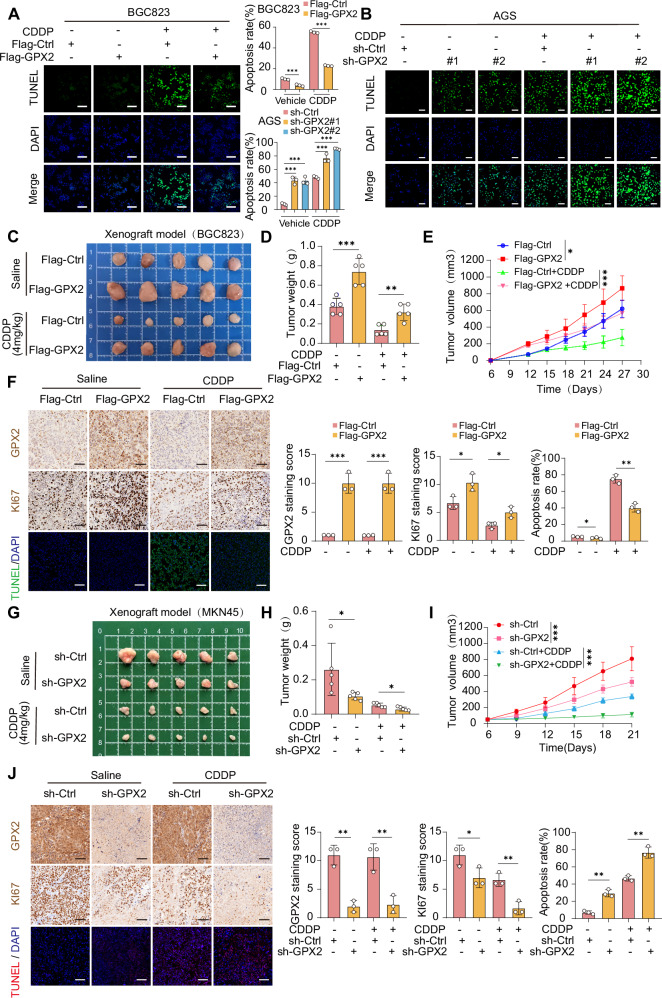
Elevated expression of GPX2 can counteract apoptosis induced by cisplatin and diminish drug sensitivity. **A**, **B** GPX2-overexpressed BGC823 cells (0.8 μg/ml) and GPX2-knockdown AGS cells (1.6 μg/ml) were treated with cisplatin for 24 h. Apoptosis levels were assessed using the TUNEL assay. Scale bars = 100 μm. **C**–**F** BGC823 cell-derived subcutaneous xenografts. **C** Representative images of subcutaneous tumors from each group. **D** Graph showing the tumor weights at the end of the experiment. **E** Tumor growth curves, with tumor volumes, are monitored every 3 days. **F** Immunohistochemistry (IHC) was used to assess the expression of GPX2 and Ki67 in subcutaneous tumors of each group. Scale bars = 50 μm. The apoptosis levels in the subcutaneous tumors of each group were evaluated using the TUNEL assay. Scale bars = 100 μm. **G**–**J** MKN45 cell-derived subcutaneous xenografts. **G** Representative images of subcutaneous tumors from each group. **H** Graph showing the tumor weights at the end of the experiment. **I** Tumor growth curves, with tumor volumes, are monitored every 3 days. **J** Immunohistochemistry (IHC) was used to assess the expression of GPX2 and Ki67 in subcutaneous tumors of each group. Scale bars = 50 μm. The apoptosis levels in the subcutaneous tumors of each group were evaluated using the TUNEL assay. Scale bars = 100 μm. The data are represented as the mean ± SD of three independent experiments. * *p* < 0.05; ** *p* < 0.01; *** *p* < 0.001, ns no significance.

Cisplatin, a platinum-based chemotherapeutic agent, is widely used to treat GC and other malignancies due to its ability to induce DNA damage and tumor cell apoptosis [41, 42]. In previous studies, we observed that the DNA repair pathway was significantly enriched in cells with high GPX2 expression, indicating an enhanced DNA repair capacity that contributes to cisplatin resistance. Given these findings, we hypothesized that GPX2 downregulation could increase the susceptibility of GC cells to cisplatin-induced apoptosis. TUNEL assays and flow cytometry analysis showed a significant increase in apoptotic cell counts following cisplatin treatment, with associated changes in apoptotic markers—increased BAX and c-PARP1 expression, and decreased BCL2 expression. However, these effects were notably reversed in GPX2-OE cells (Fig. 8A and Supplementary Fig. 12A, C, E, H), suggesting that GPX2 plays a protective role against cisplatin-induced apoptosis. Further investigations in GPX2-knockdown cells supported these results, demonstrating enhanced sensitivity to cisplatin-induced apoptosis (Fig. 8B and Supplementary Fig. 12B, D, F, G).

To assess the broader implications of GPX2 on tumor growth and cisplatin efficacy, we employed a xenograft model using BGC823 cells. Our in vivo studies revealed that GPX2 overexpression significantly accelerated tumor growth compared to controls (Fig. 8C–E). While cisplatin treatment generally suppressed tumor growth, this effect was substantially mitigated in tumors with GPX2 overexpression. Enhanced expression of the proliferation marker Ki-67 in the GPX2-overexpressing tumors versus controls and a marked reduction in apoptotic cells observed through TUNEL assay (Fig. 8F) further confirmed the antagonistic effect of GPX2 on cisplatin-induced apoptosis. Additionally, we conducted a in vivo experiment to further explore the efficacy potential of combination therapy with cisplatin and GPX2 inhibition. In a xenograft model of MKN45 cells, compared with the control group, we observed that knockdown of GPX2 inhibited tumor growth and enhanced cisplatin drug sensitivity, further confirming the synergistic promotion of GPX2 knockdown on cisplatin-induced apoptosis (Fig. 8G–J). In summary, these results collectively indicate that GPX2 may serve as a novel target for overcoming GC cisplatin resistance.

## Discussion

DGC is distinguished by poor cell cohesion, aggressive invasion of the extracellular matrix, and low cell differentiation, leading to early metastasis, primarily to the peritoneum, and resulting in a significantly reduced survival rate. To date, few signatures specific to DGC subtypes have been identified. The CDH1 gene, encoding E-cadherin, is the most recognized gene linked to hereditary DGC [43]. Genomic studies of DGC have shown that RHOA mutations are common in this subtype but rare in other GC types [44]. Li et al. noted that KRT17 loss causes cytoskeletal reorganization, activating the YAP signaling pathway and upregulating IL6 expression, thus promoting GC cell metastasis [29]. In this study, we used bioinformatics analysis of single-cell data from the public GEO database to identify markers associated with DGC progression. Our analysis showed significant overexpression of GPX2 in the malignant epithelial cells of DGC. This finding was further validated in poorly differentiated and highly malignant GC cell lines. Survival analysis indicated that elevated GPX2 expression was closely associated with poor prognosis in gastric cancer patients. Functional validation experiments confirmed that GPX2 enhances the proliferation, migration, and invasion of DGC cells. Based on our findings, we suggest that GPX2 is a potential biomarker for DGC prognosis and a promising therapeutic target.

There is growing evidence supporting the intricate relationship between mitochondrial homeostasis and lipid metabolism. For instance, CES2 disrupts lipid homeostasis, leading to mitochondrial dysfunction in oral squamous cell carcinoma [45]. Arf1 directs the flow of fatty acids and metabolites from lipid droplets to peroxisomes and mitochondria, thereby regulating mitochondrial function [46]. DGAT1-dependent lipid droplet biogenesis protects against lipotoxic mitochondrial damage [24]. GPX2 impacts mitochondrial homeostasis by modulating lipid metabolism and lipid droplet production. Additionally, lipid droplet accumulation can stabilize the endoplasmic reticulum (ER), mitigate ER stress, and promote tumor proliferation and metastasis. upregulation of ACSS3 in prostate cancer cells regulates PLIN3 stability, reduces lipid droplet deposition, and induces ER stress-mediated apoptosis, reversing enzalutamide resistance and hindering castration-resistant prostate cancer progression [47]. Our study shows that inhibiting GPX2 exacerbates ER stress in GC cells by disrupting lipid homeostasis. Given that mitochondrial dysfunction and ER stress are pivotal pathways for inducing apoptosis. LPCAT2-mediated lipid droplet production has been shown to confer resistance to 5-fluorouracil and oxaliplatin in colorectal cancer cells [48]. Similarly, an increase in FASN-mediated de novo lipogenesis is associated with gemcitabine resistance in pancreatic cancer [49]. In our study, by identifying GPX2 as a key regulator of lipid synthesis and an apoptosis inducer through various mechanisms, we further confirmed that high GPX2 expression counteracts cisplatin-induced apoptosis and promotes tumor chemoresistance. Targeting GPX2 could be an effective therapeutic strategy to enhance cisplatin sensitivity in advanced DGC patients, potentially improving their prognosis.

Specific lipid profiles have emerged as unique biomarkers with diagnostic, therapeutic, and prognostic potential [50]. Increasing studies have emphasized the use of LC-MS/MS-based prediction and comprehensive statistical evaluation of LPC parameters to quantify liposome targeting strategies, thereby quantifying LPCs as evaluable novel cancer biomarkers [51]. In the present study, liposome sequencing demonstrated that GPX2 silencing resulted in significantly decreased triglyceride and increased acylcarnitine triglyceride levels. Acylcarnitine plays an important role in transporting fatty acids into mitochondria for beta-oxidation. Dysregulation of acylcarnitine metabolism is associated with various diseases, such as myocardial ischemia, neurodegenerative diseases, and diabetes [52]. Recent studies have also highlighted the potential anti-tumor effects of acylcarnitine in cancers such as liver, prostate, and colon cancer [53–55]. However, the function and the specific mechanism of acylcarnitine in GC remain unclear. Previous studies have shown that palmitoylcarnitine can reduce OXPHOS-dependent mitochondrial respiration, induce mitochondrial membrane hyperpolarization, and increase the production of reactive oxygen species. The accumulation of long-chain acylcarnitine exhibits a negative impact on mitochondrial function and may lead to myocardial energy crises during ischemia and reperfusion [52, 56]. Furthermore, reduced CPT1 levels have been shown to cause mitochondrial dysfunction and impaired fatty acid oxidation and worsen renal fibrosis through increased acylcarnitine accumulation [57]. Our findings confirm that GPX2 inhibition induces apoptosis in GC cells by elevating acylcarnitine levels and impairing mitochondrial function. Acylcarnitine could emerge as a novel metabolic marker for DGC with GPX2 playing a crucial role in its synthesis and metabolism.

While our findings establish GPX2 as a promising therapeutic target for overcoming cisplatin resistance in gastric cancer, clinical translation faces significant challenges. Both small molecule inhibitors and RNAi-based therapies encounter delivery barriers, where small molecules require optimized bioavailability and tumor penetration while RNAi necessitates advanced delivery systems such as nanoparticles or viral vectors to protect against degradation and ensure targeted delivery [58–60]. Furthermore, off-target effects remain a concern as small molecules may inhibit related GPX family members and RNAi could silence genes with sequence similarities. Potential on-target toxicity in normal tissues where GPX2 provides essential antioxidant protection also warrants consideration. These limitations could be addressed through developing isoform-specific inhibitors and rational combination strategies with existing therapies to enhance safety and efficacy.

In summary, we conceive that GPX2 is an important oncogenic factor that promotes the malignant progression of DGC and correlates with poor prognosis. Targeting GPX2 facilitates the reprogramming of lipid metabolism, leading to elevated acylcarnitine levels that impair mitochondrial function. This effect works together with endoplasmic reticulum stress to induce apoptosis in GC cells and inhibit tumor growth. Importantly, we demonstrated the role of GPX2 in cisplatin resistance and its potential as a chemotherapeutic target for DGC. Our findings indicate that GPX2 is a promising target for anti-tumor therapy and overcoming cisplatin resistance in DGC, presenting a novel strategy for targeting lipid metabolism in GC therapy.

## Materials and methods

### Cell lines and cell culture

The AGS GC cell lines were purchased from the American Type Culture Collection (ATCC, MD, USA), and human GES1 cells and the BGC823, and HGC27 GC cell lines were obtained from the Type Culture Collection of the Chinese Academy of Sciences (TCCCAS, Shanghai, China). SNU601, SNU216, and MKN45 cells were purchased from the Korean Cell Line Bank (KCLB, Seoul, Korea). MKN1and MKN74 cells were purchased from the JCRB Cell Bank (JCRB, Osaka, Japan). All cells were cultured in RPMI 1640 medium (Gibco, CA, USA), containing 10% Fetal bovine serum (FBS, Gibco, CA, USA), penicillin (100 U/mL), and streptomycin (100 μg/mL) (Invitrogen, MA, USA) at 37 °C in humidity of 5% CO_2_.

### Human gastric cancer specimen

The study utilized 160 pairs of GC tissue embedded in tissue microarrays and 10 pairs of fresh tumor and adjacent non-cancerous tissue samples. These specimens were provided by the Department of Gastroenterology at Nanjing Drum Tower Hospital. Informed consent was obtained from all patients for the use of these samples. All experiments involving human specimens were approved by the Medical Research Ethics Committee of Nanjing Drum Tower Hospital.

### Animal models

Male BALB/c nude mice (5–6 weeks, GemPharmatech, Nanjing, China) and maintained in SPF conditions. Groups of mice (*n* = 5 per group) received axillary inoculations with either 3 × 10^6^ BGC823 cells alone or cells transfected with GPX2-OE or control vector. Tumor volumes were regularly assessed using the formula (volume = length × width^2^ × 1/2). Treatment commenced once the volume of any subcutaneous tumor exceeded 100 mm^3^, involving thrice-weekly intraperitoneal injections of either cisplatin 3 mg/kg (MCE, NJ, USA) for the treatment group or normal saline for the control group. After four weeks, the mice were euthanized, and their tumors were harvested and subjected to either fixation in 4% paraformaldehyde or preservation by freezing. MKN45 cells (5 × 10^5^ cells/mouse, in 100 μL 1:1 mixture of PBS and Matrigel) were subcutaneously injected into the right flank of nude mice. The drug administration and data collection methods were identical to those previously described. The experiment was terminated approximately 3 weeks later.

### siRNA and lentiviral transduction

siRNAs targeting GPX2 were designed and synthesized by RiboBio (Guangzhou, China). The siRNA sequences for GPX2 knockdown are as follows: si-GPX2#1:5′-ACATCAAGCGCCTCCTTAA-3′ or si-GPX2#2: 5′-CCCTTATGATGACCCATTT-3′. shRNAs targeting GPX2 were designed based on siRNA sequences. GPX2 lentiviruses were constructed by Corues Biotechnology (Nanjing, China) using VP004-CMV-MCS-3flag-EF1-fLUC-T2A-PURO vectors. siRNA was transfected into cells (AGS and SNU601) with Lipofectamine 3000 (Invitrogen, CA, USA). GPX2-OE lentivirus and their vectors were added to BGC823 and HGC27 cells. After 72 h, infected cells were selected with 2 μg/mL puromycin (Sigma, MO, USA).

### Immunohistochemistry (IHC)

The microarray slides were processed and stained by Servicebio (Wuhan, China), following a standardized staining protocol. The staining intensity of GPX2 was independently evaluated by two pathologists, who were blinded to the clinical data, using a semi-quantitative immunoreactivity score (IRS).

### Quantitative real-time PCR

Total RNA from tissues or cells was extracted using Trizol reagent (Invitrogen, CA, USA) and reverse-transcribed into cDNA using a reverse transcription kit (Vazyme, Nanjing, China). Subsequently, real-time quantitative PCR (qPCR) analysis was performed using ChamQ SYBR Color qPCR Master Mix (Vazyme, Nanjing, China), following the manufacturer’s instructions strictly. The expression level of the target gene was normalized to actin, which served as the internal control. The primer sequences for specific genes are as follows: GPX2-Forward: GGTAGATTTCAATACGTTCCGGG; GPX2-Reverse: TGACAGTTCTCCTGATGTCCAAA; β-actin-Forward: CATGTACGTTGCTATCCAGGC; β-actin-Reverse: CTCCTTAATGTCACGCACGAT.

### Western blot

The proteins were analyzed by Western blotting, following established protocols [61]. The primary antibodies used were as follows: β-actin (Proteintech, 23660-1-AP); GPX2 (Abcam, ab137431); ACC (HUABIO, RT1015); FASN (HUABIO, R1706-8); ACLY (HUABIO, R1706-75); BAX(CST, 9548T); BCL2(PTM BIO, A18415); c-PARP1 (PTM BIO, PTM-7466); GRP78/BIP (ABclonal, A0241); p-eIF2α (CST, 3597S); p-PERK (ABclonal, AP0086); e-IRE1a (ABclonal, AP1146); XBP1s (ABclonal, A17007).

### Proliferation assay

#### Colony formation

Following exposure to various conditions, 1000 cells per well were seeded into a six-well culture plate and incubated for two weeks. Subsequently, the cells were fixed in methanol for 15 min and stained with crystal violet for 10 min. Colonies comprising more than 50 cells were quantified using ImageJ software [62].

#### CCK8 assay

The treated cells were seeded into 96-well plates at a density of 3,000 cells per well and incubated at 0, 24, 48, 72, and 96 h. To assess cell viability, 100 µL of CCK-8 working solution (Vazyme, Nanjing, China). The plates were incubated at 37 °C, and the optical density (OD) at 450 nm was measured every 30 min using a microplate reader.

### Transwell assay

The migratory and invasive capabilities were assessed using Transwell chambers (Corning, MA, USA). A suspension of 5 × 10^4^ cells per well in serum-free medium was inoculated into the upper chamber, either with or without 50 μg of matrix gel (Corning, MA, USA). The lower chamber was filled with 500 μL of culture medium containing 20% serum. After a 24-h incubation at 37 °C, the cells were fixed for 20 min in 4% paraformaldehyde and stained for 30 min with crystal violet (Beyotime, Shanghai, China). Finally, three visual fields were selected under the microscope and quantified using Photoshop [63].

### Oil Red O staining and Bodipy 493/503 staining

The cells were treated with 200 μM OA (Sigma, MO, USA) for 24 h. For Oil Red O staining: Oil Red O powder (Sigma, MO, USA) was diluted with distilled water at a 3:2 ratio, then filtered to prepare the working solution. Samples were incubated in 60% isopropanol for 15 min, stained with the Oil Red O working solution for 10 min, and subsequently stained with hematoxylin. For BODIPY493/503 staining: 100 μL of a 10 μM BODIPY 493/503 working solution (MCE, NJ, USA) was added to each well for a staining duration of 30 min. Subsequently, the nuclei were stained with DAPI and imaged using a Leica microscope.

### TUNEL assay

Cell apoptosis was detected using a TUNEL Apoptosis Detection Kit (KeyGEN BioTECH, Nanjing, China). Cell nuclei were stained with DAPI. Confocal images of cells were sequentially acquired on a Leica Thunder Imaging Microscope System, and the number of TUNEL-positive cells was counted.

### ER tracker assay

After treatment, the cells were washed with PBS and stained with ER-Tracker Red working solution (Beyotime Biotechnology, Nanjing, China), followed by incubation for 15 to 30 min at 37 °C. Subsequently, DAPI was added as an anti-fluorescence quenching agent.

### Electron microscopy

Cells from each treatment group were fixed in 2.5% glutaraldehyde, centrifuged, fixed for 10 min at room temperature, and stored at 4 °C. The samples were processed according to the standard embedding and sectioning protocols for transmission electron microscopy by Biossci Biotechnology Co., Ltd. (Wuhan, China). The resulting images were collected and evaluated by expert pathologists.

### Lipidomics analysis

1 × 10^6^ AGS cells transfected with shCtrl or shGPX2 lentivirus were centrifuged at 1500 rpm for 10 min, after which the cell pellets were frozen in liquid nitrogen. The samples were then sent to Shanghai Majorbio Bio-Pharm Technology Co., Ltd. for non-targeted liquid chromatography-mass spectrometry (LC-MS) analysis.

### Flow cytometry

#### Reactive oxygen species detection

The stock solution of 10 mM DCFH-DA (Keygen Biotech, Nanjing, China) was diluted with PBS to create a 10 μM working solution. Each sample was incubated at 37 °C for 20 min, and flow cytometry analysis was performed within one hour after incubation.

#### Cell apoptosis detection

Supernatant and adherent cell samples were collected and re-suspended in 500 µL of Binding Buffer. Each sample was then gently mixed with 5 µL of Annexin V-FITC and 5 µL of propidium iodide (Beyotime Biotech, Nanjing, China). The samples were incubated in the dark at room temperature for 10 min and then analyzed by flow cytometry.

#### JC-1 staining and JC-1 detection

Prepare the JC-1 working solution according to the provided instructions. Add 1 mL of JC-1 staining solution to each sample and incubate at 37 °C for 20 min. After incubation, wash the cells with 1× JC-1 staining buffer. The cells can then be observed under a confocal microscope or analyzed by flow cytometry.

### Statistical analysis

We accessed public DGC single-cell datasets and analyzed the single-cell expression data using Seurat version 4.3.0. We adhered to the standard comparative analysis workflow, consistent with our previously established research methodologies [61]. All experiments were performed in at least three independent biological replicates, each with technical replicates, and data were presented as mean ± SD. Statistical analyses were carried out using GraphPad Prism 8.0 software, with *P* < 0.05 considered statistically significant. All *P* values were indicated in the figures (ns: *P* > 0.05, *: *P* < 0.05, **: *P* < 0.01, ***: *P* < 0.001).

## Supplementary information

Supplementary data

Orignal data

## Data Availability

Public bull RNA-seq datasets and single-cell RNA-seq data used for this analysis were downloaded from the GEO data repository (GSE62254, GSE183904 and GSE167297) and TCGA-STAD database.
