# Supplementary material for: Targeting GPX2 to disrupt lipid homeostasis and enhance cisplatin sensitivity in diffuse gastric cancer
Source: Cell Death Discov. 2025 Oct 27;11:491. doi: 10.1038/s41420-025-02771-8 (PMC12559244; doi:10.1038/s41420-025-02771-8)
Supplement: Supplementary file 1 — Supplementary data [file 41420_2025_2771_MOESM1_ESM.pdf]

Supplementary data

Supplementary Table 1: Clinical and pathologic features of the DGC sample used in our study.

Number	GEO dataset	Patient ID	Age	Gender	Laurens	Tumor	Normal
**DGC1**	GSE183904	NGCII510	66	F	Diffuse	Y	
**DGC2**	GSE183904	NGCII519	83	M	Diffuse	Y	
**DGC3**	GSE183904	NGCII520	83	M	Diffuse	Y	
**DGC4**	GSE183904	NGCII531	70	F	Diffuse	Y	
**DGC5**	GSE183904	NGCII545	60	M	Diffuse	Y	
**DGC6**	GSE167297	p1	65	F	Diffuse	Y	
**DGC7**	GSE167297	p2	69	M	Diffuse	Y	
**DGC8**	GSE167297	p3	60	M	Diffuse	Y	
**DGC9**	GSE167297	p4	46	F	Diffuse	Y	
**DGC10**	GSE167297	p5	59	M	Diffuse	Y	
**NC1**	GSE183904	NGCII520	83	M	Diffuse		Y
**NC2**	GSE167297	p1	65	F	Diffuse		Y
**NC3**	GSE167297	p3	60	M	Diffuse		Y
**NC4**	GSE167297	p4	46	F	Diffuse		Y
**NC5**	GSE167297	p5	59	M	Diffuse		Y

Supplementary Table 2: Baseline data were collected from 160 gastric cancer patients in tissue microarray.

Characteristics	Low<br>N=80	High<br>N=80	P value
Age, n (%)			0.629
≤65	46 (28.7%)	49 (30.6%)	
＞65	34 (21.2%)	31 (19.4%)	
Gender, n (%)			0.273
Male	63 (39.4%)	57 (35.6%)	
Female	17 (10.6%)	23 (14.4%)	
TNM stage, n (%)			0.483
I	7 (4.4%)	12 (7.5%)	
II	22 (13.8%)	21 (13.1%)	
III	51 (31.9%)	46 (28.7%)	
IV	0 (0%)	1 (0.6%)	
T, n (%)			0.111
T1	4 (2.5%)	11 (6.9%)	
T2	12 (7.5%)	13 (8.1%)	
T3	35 (21.9%)	38 (23.8%)	
T4	29 (18.1%)	18 (11.2%)	
N, n (%)			0.923
N0	15 (9.4%)	16 (10%)	
N1	15 (9.4%)	18 (11.2%)	
N2	18 (11.2%)	17 (10.6%)	
N3	32 (20%)	29 (18.1%)	
M, n (%)			0.477
M0	80 (50%)	78 (48.8%)	
M1	0 (0%)	2 (1.2%)	
Lauren, n (%)			0.838
IGC	37 (25.2%)	33 (22.4%)	
DGC	19 (12.9%)	20 (13.6%)	
Mixed	18 (12.2%)	20 (13.6%)	

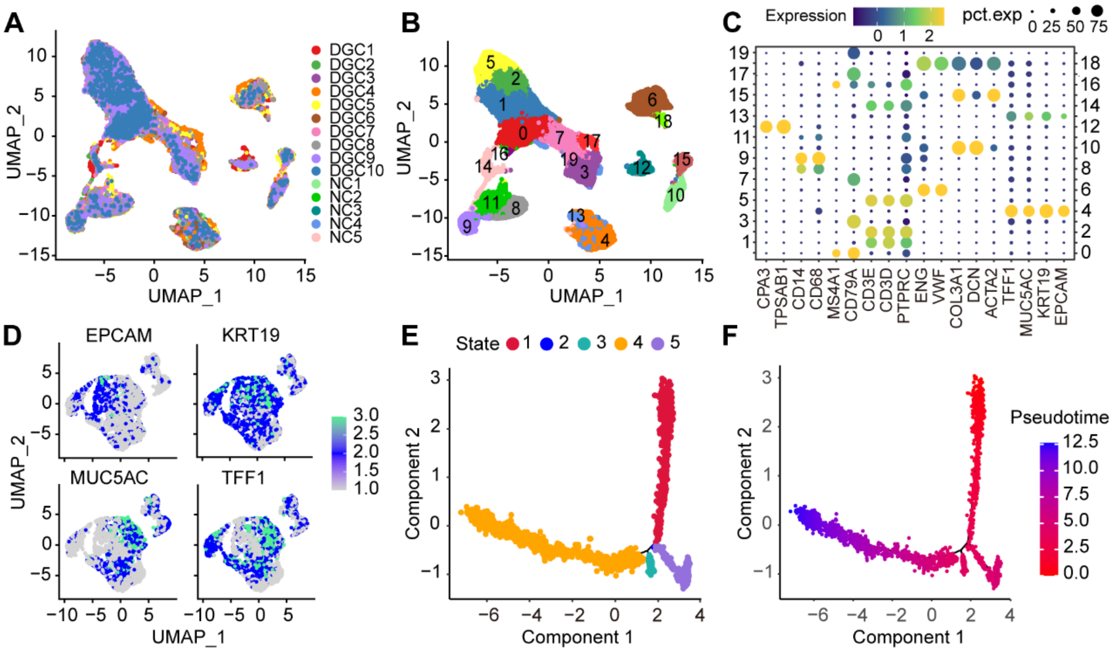

Supplementary Fig.1. The single-cell landscape of DGC. (A) UMAP of DGC and Normal Samples. (B)UMAP representation shows the division of all cells into 20 clusters after dimensionality reduction and clustering. (C) Dot plot illustrating the expression of representative marker genes for each cell type in the 20 cell clusters. (D) UMAP analysis reveals the expression patterns of epithelial markers in gastric epithelial cells. (E-F) Pseudotime analysis of cell progression.

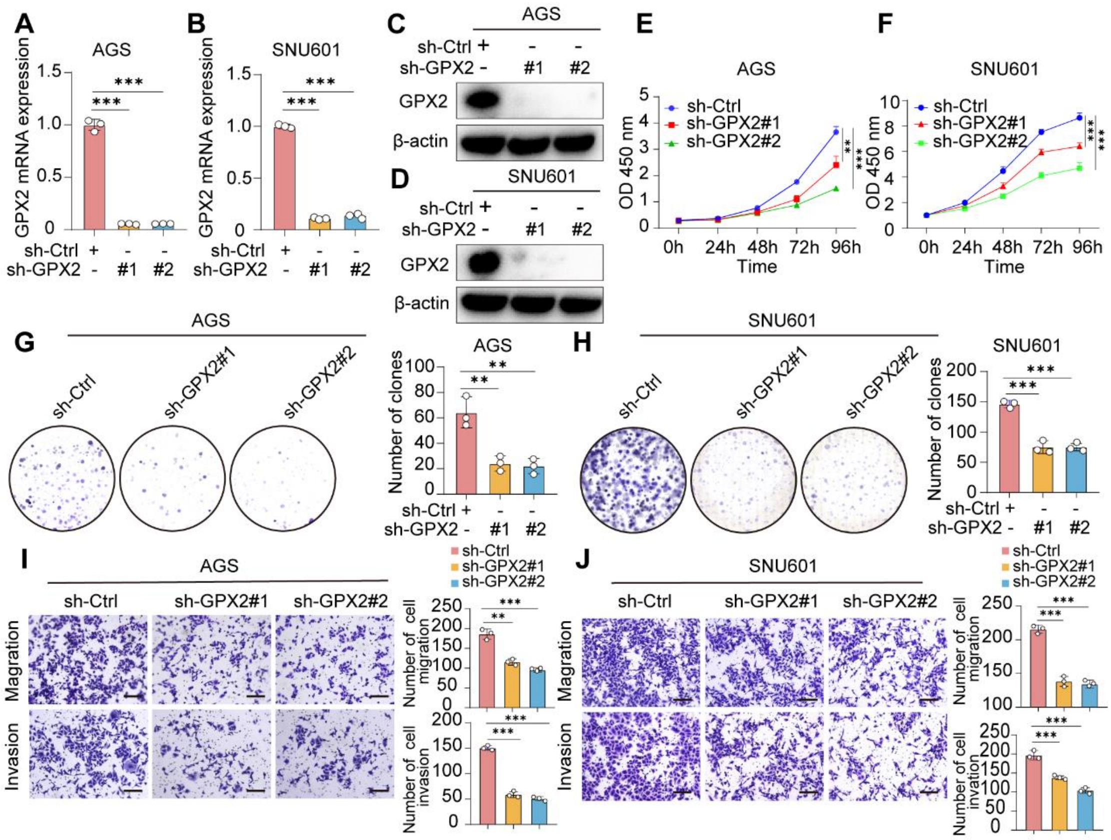

Supplementary Fig.2. Silencing GPX2 expression suppresses the proliferation, invasion, and migration of GC cells. (A-B) Quantitative real-time PCR (qRT-PCR) was employed to assess the knockdown efficiency of the GPX2-targeting shRNA in AGS and SNU601 cells. (C-D) Western blot analysis was also conducted to evaluate the knockdown efficiency of the GPX2 shRNA in AGS and SNU601 cells. (E-F) The CCK-8 assay was utilized to investigate the impact of GPX2 silencing on the proliferation of AGS and SNU601 cells. (G-H) The effect of GPX2 knockdown on the proliferation of AGS and SNU601 cells was confirmed by a colony formation assay. (I-J) The Transwell assay was employed to assess the impact of GPX2 knockdown on the invasion and migration capabilities of AGS and SNU601 cells. Scale bars = 200 μm. The data are represented as the mean ±SD of three independent experiments. * $p < 0.05$; ** $p < 0.01$; *** $p < 0.001$, ns, no significance.

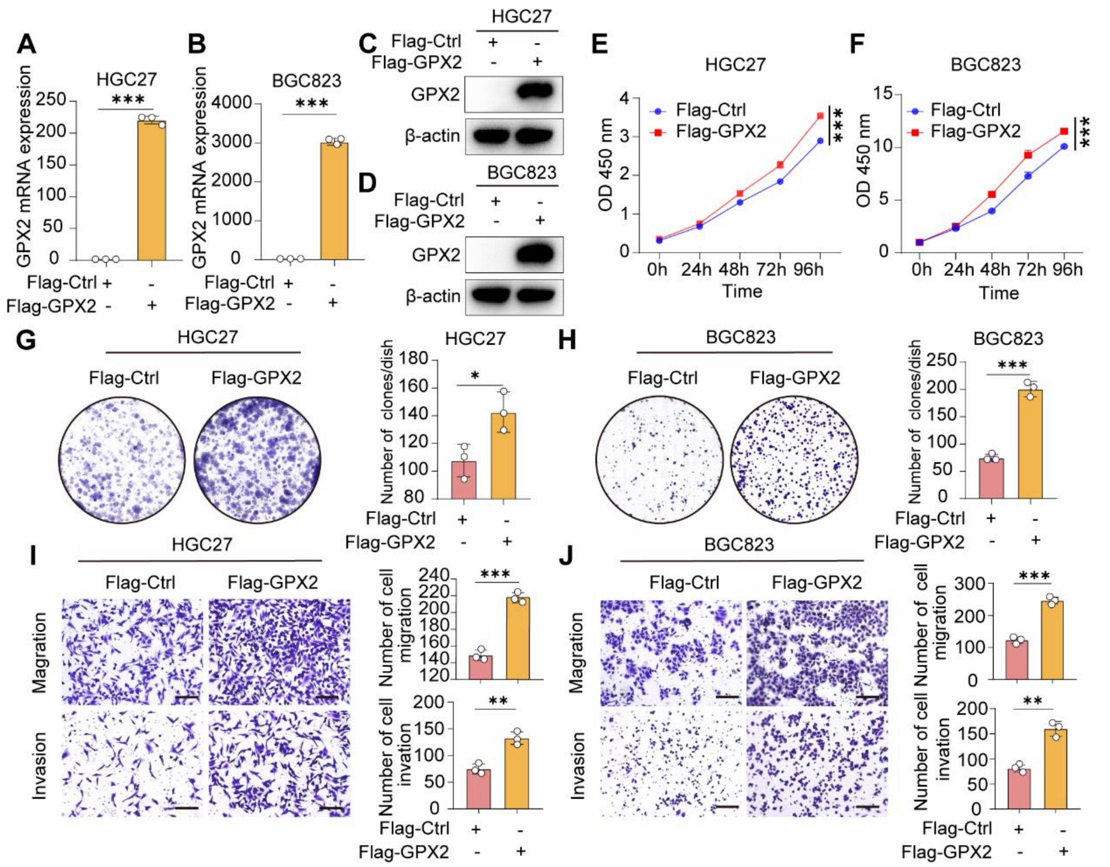

Supplementary Fig.3. Overexpression of GPX2 enhances the proliferation, invasion, and migration of gastric cancer cells. (A-B) Quantitative real-time PCR (qRT-PCR) was employed to assess the overexpression efficiency of the GPX2-overexpressing virus in BGC823 and HGC27 cells. (C-D) Western blot analysis was utilized to evaluate the overexpression efficiency of the GPX2-overexpressing virus in BGC823 and HGC27 cells. (E-F) The impact of GPX2 overexpression on the proliferation of BGC823 and HGC27 cells was assessed using the CCK-8 assay. (G-H) The effects of GPX2 overexpression on the proliferation of BGC823 and HGC27 cells were evaluated using a colony formation assay. (I-J) The effects of GPX2 overexpression on the invasion and migration abilities of BGC823 and HGC27 cells were assessed using Transwell assays. Scale bars = 200 μm.The data are represented as the mean ±SD of three independent experiments. * $p < 0.05$; ** $p < 0.01$; *** $p < 0.001$, ns, no significance.

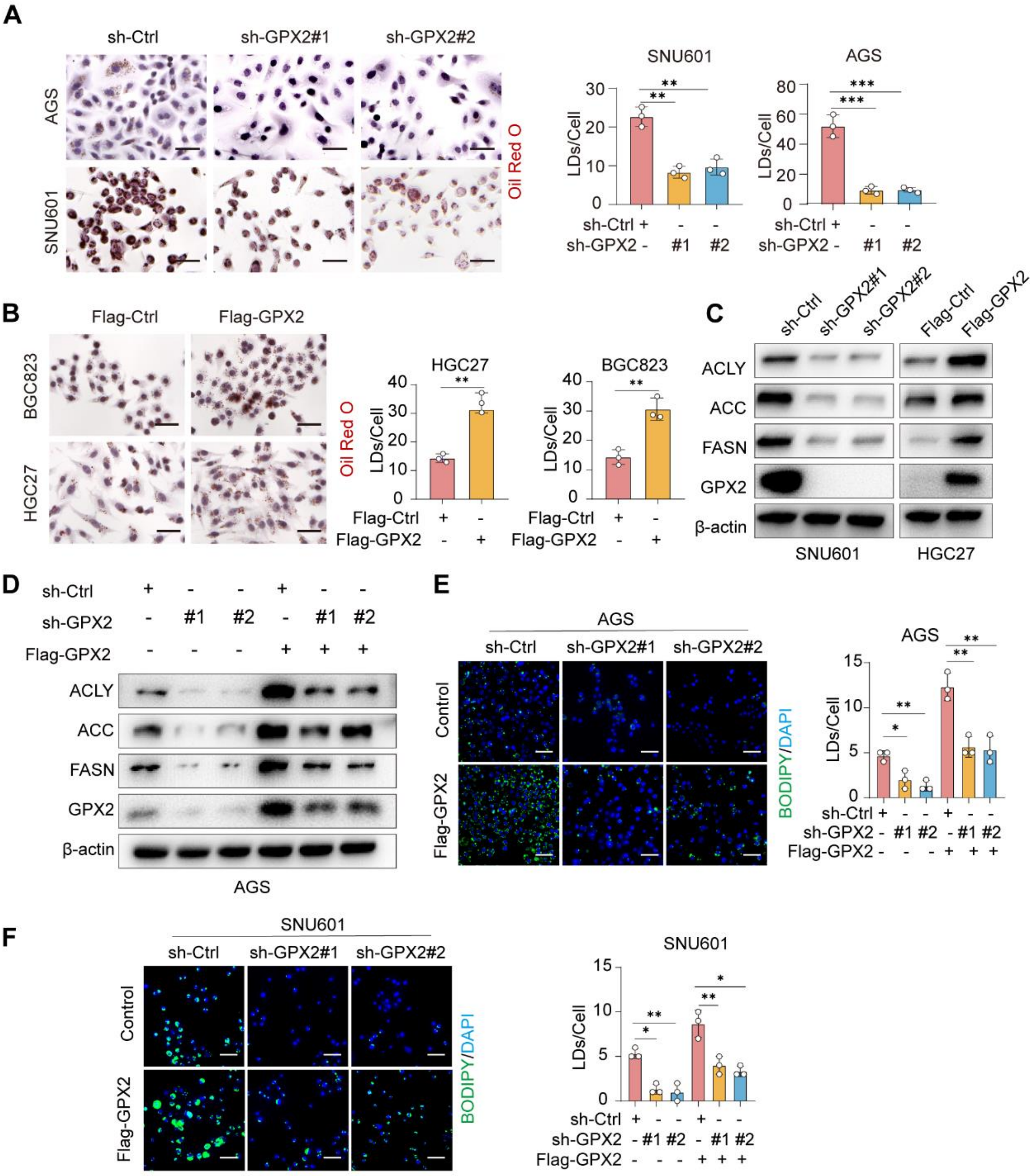

Supplementary Fig.4. GPX2 enhances lipid droplet formation and regulates lipid synthesis in gastric cancer cells. (A)Oil Red O staining of GPX2-knockdown AGS and SNU601 cells, induced by 200 μM oleic acid for 24 hours, showing lipid droplet formation. Scale bars = 50 μm. (B) Lipid droplet formation in BGC823 and HGC27 cells transfected with either control virus or GPX2-overexpressing virus was induced with 200 μM oleic acid for 24 hours and visualized by Oil Red O staining. Scale: 50 μm. (C) The protein levels of

lipid metabolism-related genes in GPX2-knowdown SNU601 and overexpressed-GPX2 HGC27 cells were assessed by Western blot analysis. (D) Western blot analysis of lipid metabolism related gene expression in control and GPX2 knockdown AGS after re-overexpressing GPX2. (E-F) GPX2 was overexpressed in control and GPX2 knockdown AGS and SNU601 cells , lipid droplet formation was induced with 200μM oleic acid for 24 hours, and visualized by BODIPY493/503 fluorescent staining. Scale: 50 μm. The data are represented as the mean ±SD of three independent experiments. * $p < 0.05$; ** $p < 0.01$; *** $p < 0.001$, ns, no significance.

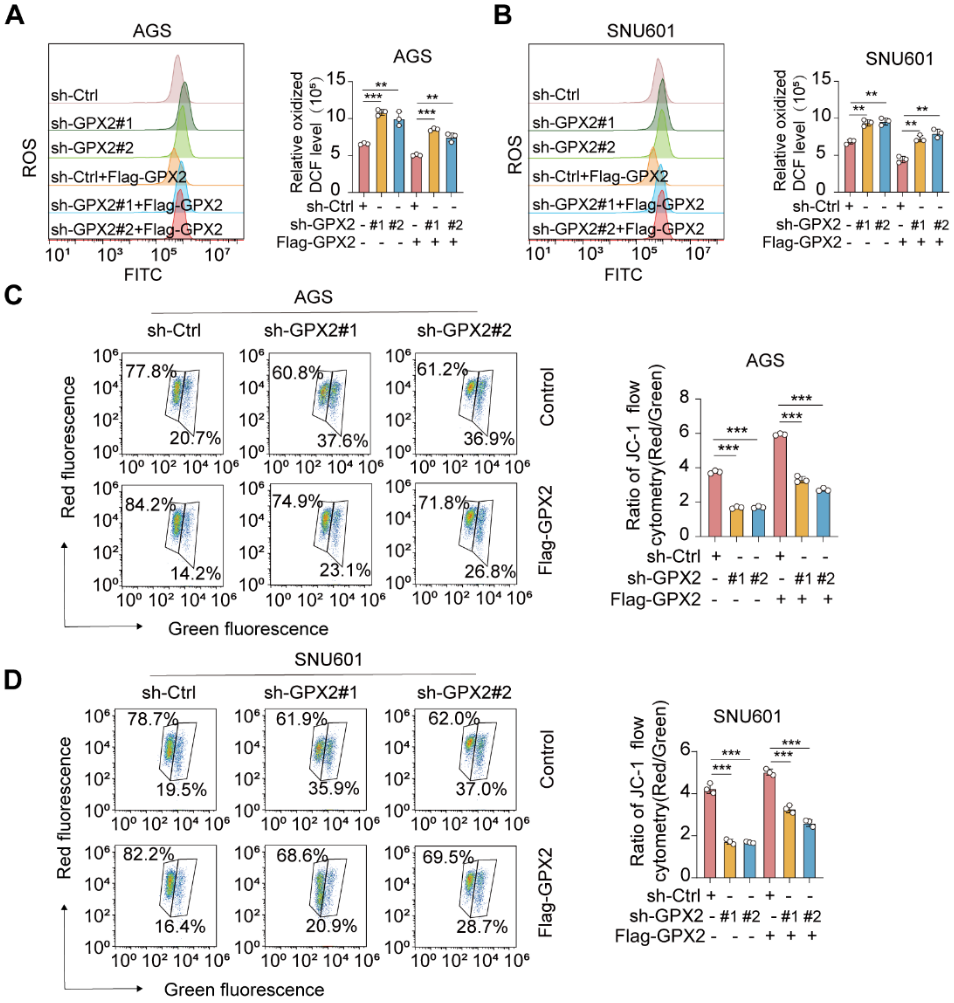

Supplementary Fig.5. GPX2 overexpression rescues mitochondrial dysfunction caused by GPX2 knockdown. (A-B) Reactive oxygen species levels were quantified by flow cytometry in control and GPX2-knockdown AGS and SNU601 cells with or without GPX2 overexpression. (C-D) Flow cytometry analysis of mitochondrial membrane potential (JC-1 assay) in control and GPX2-knockdown AGS and SNU601 cells with or without GPX2 overexpression. The data are represented as the mean ±SD of three independent experiments. * $p < 0.05$; ** $p < 0.01$; *** $p < 0.001$, ns, no significance.

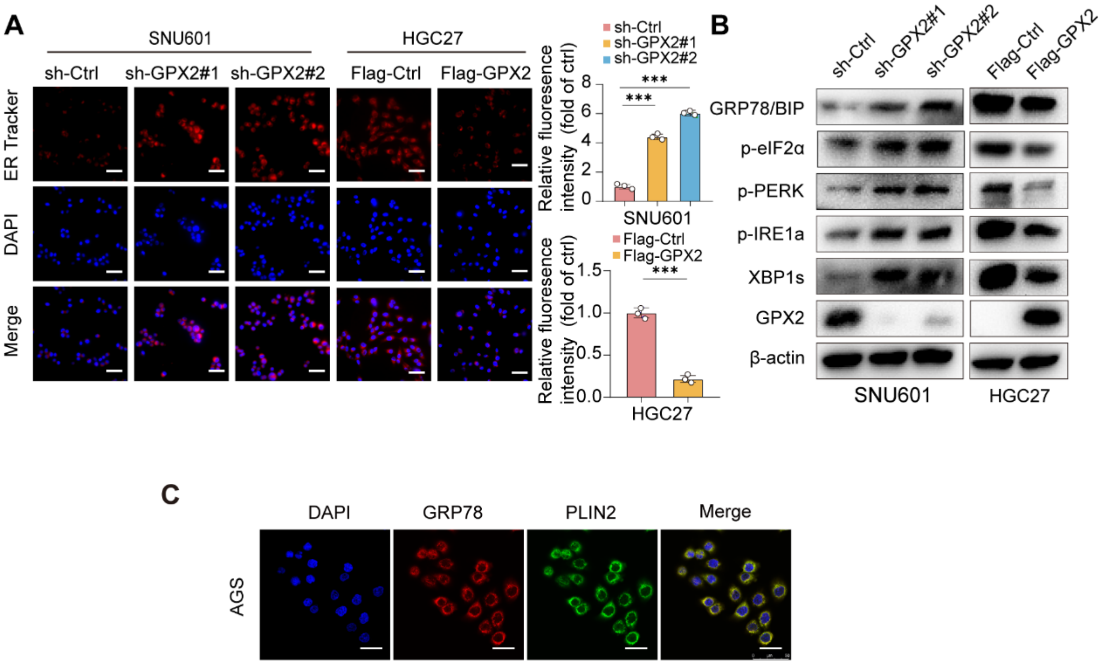

Supplementary Fig.6. GPX2 modulates endoplasmic reticulum homeostasis in gastric cancer cells. (A) ER-Tracker fluorescence staining was employed to visualize the structure of the endoplasmic reticulum in GPX2-overexpressed HGC27 cells and GPX2-knockdown SNU601. Scale bars = 50 μm. (B) Western blot analysis was utilized to assess the protein levels of endoplasmic reticulum stress-related genes in GPX2-overexpressed HGC27 cells and GPX2-knockdown SNU601. (C) Confocal microscopy analysis of GRP78 and PLIN2 co-localization in AGS gastric cancer cell. Scale bars = 50 μm. The data are represented as the mean ±SD of three independent experiments. * $p < 0.05$; ** $p < 0.01$; *** $p < 0.001$, ns, no significance.

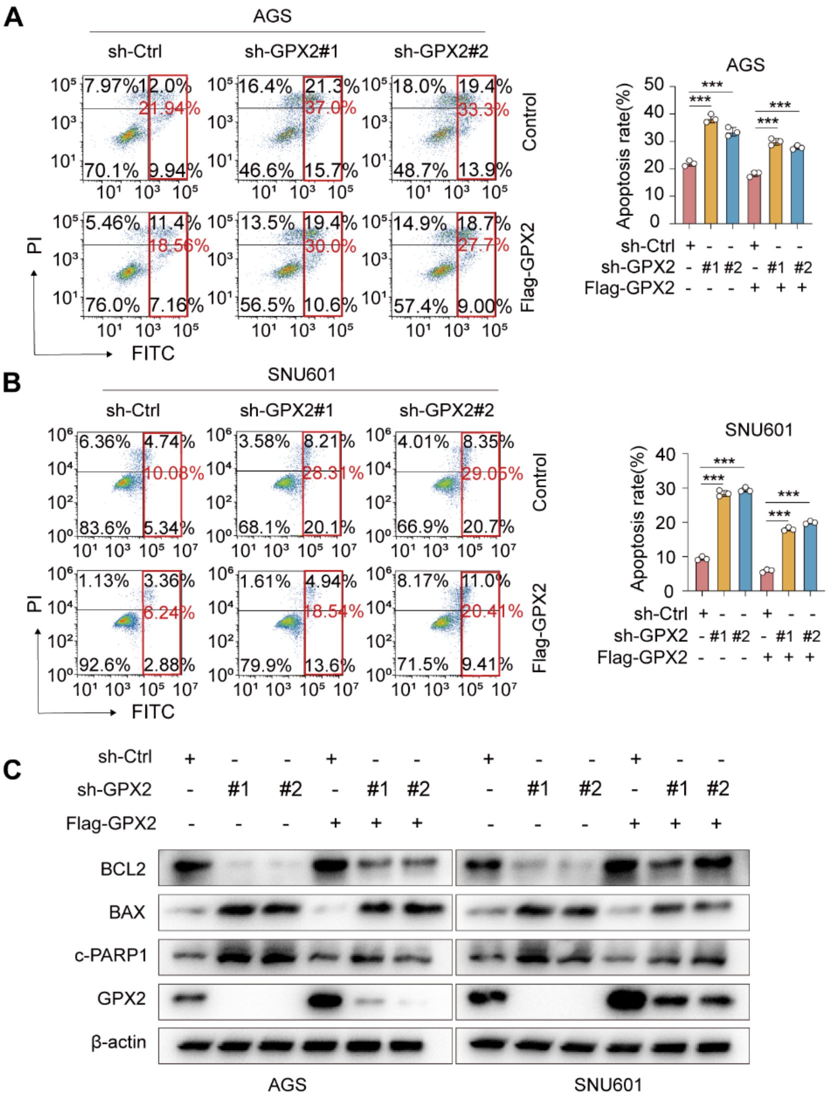

Supplementary Fig.7. GPX2 overexpression rescues cell apoptosis caused by GPX2 knockdown. (A-B) Apoptosis rates in GPX2-knockdown AGS and SNU601 cells with or without GPX2 overexpression were analyzed by flow cytometry. (C) Western blot analysis of apoptosis-related proteins in control and GPX2-knockdown AGS and SNU601 cells with or without GPX2 overexpression. The data are represented as the mean ±SD of three independent experiments. * $p < 0.05$; ** $p < 0.01$; *** $p < 0.001$, ns, no significance.

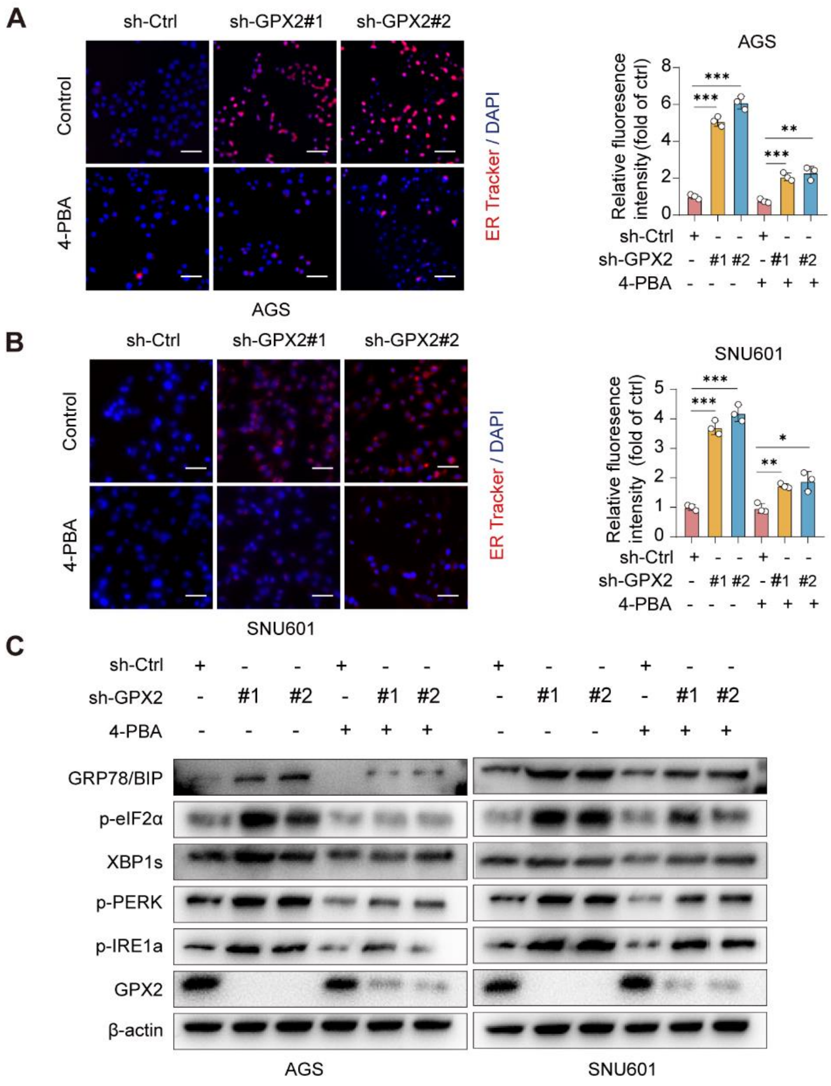

Supplementary Fig.8. 4-PBA treatment attenuated the endoplasmic reticulum stress enhancement caused by GPX2 knockdown. (A-B) ER stress analysis in GPX2-knockdown AGS and SNU601 cells treated with 10μM 4-PBA for 48 h. ER stress was visualized by ER-Tracker Red fluorescence staining. Scale bars = 50μm. (C) Western blot analysis of ER stress-related proteins in GPX2-knockdown AGS and SNU601 cells following 48 h treatment with 10μM 4-PBA. The data are represented as the mean ±SD of three independent experiments. * $p < 0.05$; ** $p < 0.01$; *** $p < 0.001$, ns, no significance.

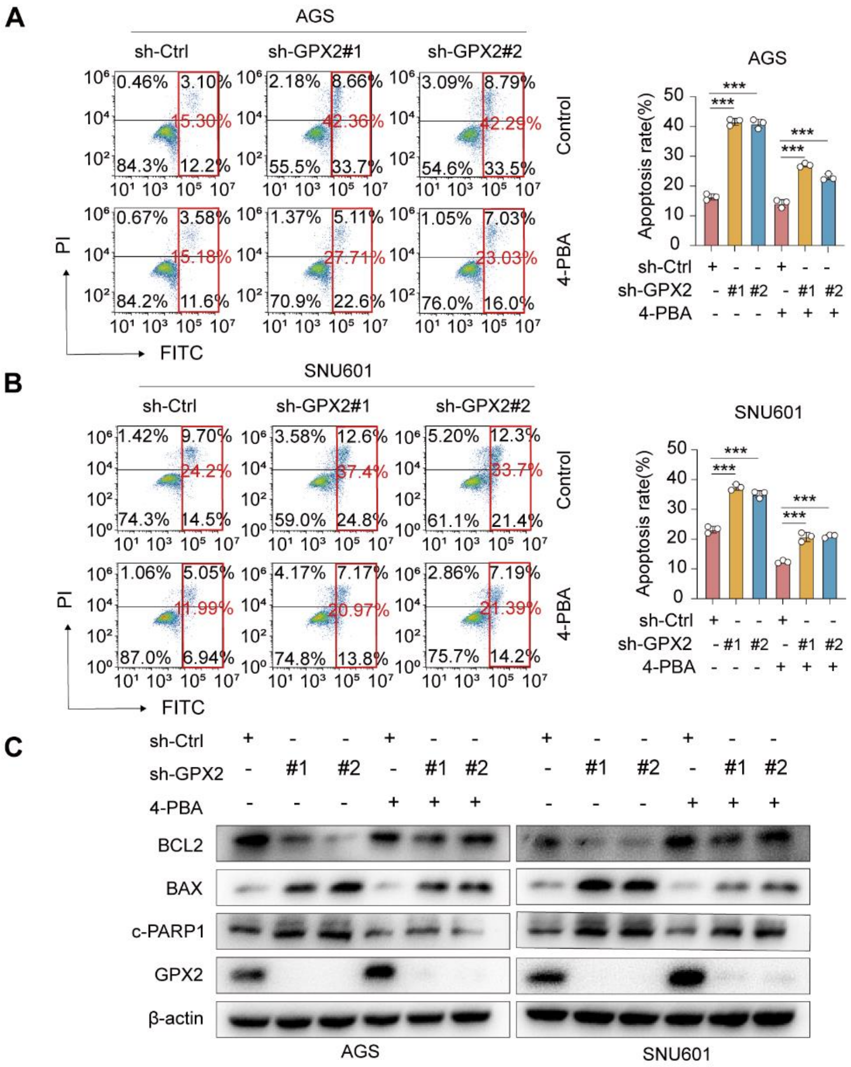

Supplementary Fig.9. 4-PBA treatment attenuated GPX2 knockdown-induced apoptosis. (A-B) Flow cytometric analysis of apoptosis rates in control and GPX2-knockdown AGS and SNU601 cells following 48 h treatment with 10μM 4-PBA. (C) Western blot analysis of apoptosis-related protein expression in control and GPX2- knockdown cells treated with 10μM 4-PBA for 48 h. The data are represented as the mean ±SD of three independent experiments. * $p < 0.05$; ** $p < 0.01$; *** $p < 0.001$, ns, no significance.

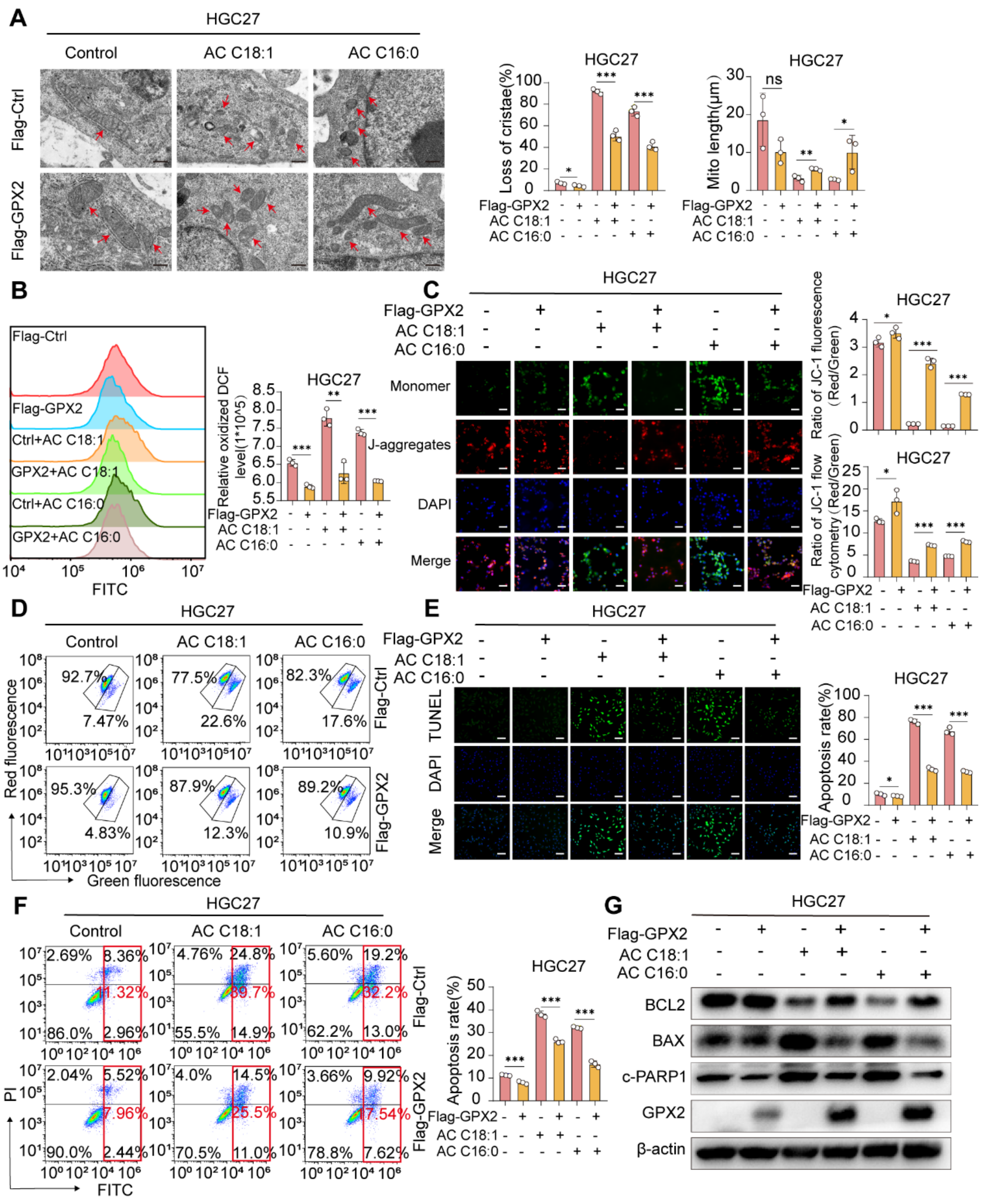

Supplementary Fig.10. GPX2 knockdown results in elevated levels of acylcarnitine, which is associated with mitochondrial dysfunction and apoptosis. HGC27 transfected with control and GPX2-overexpressing viruses, were respectively treated with 30 μM C18:1 oleoyl L-carnitine and 20 μM C16:0 palmitoyl L-carnitine for 24 hours. (A) Transmission electron microscopy revealed the structure of cell mitochondria. Red arrows indicate mitochondria. Scale bars = 500 nm. (B) Flow cytometry was utilized to detect the levels of

reactive oxygen species (ROS). (C) JC-1 fluorescence staining indicated the levels of mitochondrial membrane potential. Scale bars = 50 μm. (D) Flow cytometry was used to assess the mitochondrial membrane potential. Following treatment with 30 μM C18:1 oleoyl L-carnitine and 20 μM C16:0 palmitoyl L-carnitine for 48 hours respectively: (E) Apoptosis levels were measured by the TUNEL assay. Scale bars = 100 μm. (F) Apoptotic rates were quantified using flow cytometry. (G) Quantitative analysis of apoptosis-related protein levels was conducted using Western blot. The data are represented as the mean ±SD of three independent experiments. * $p < 0.05$; ** $p < 0.01$; *** $p < 0.001$, ns, no significance.

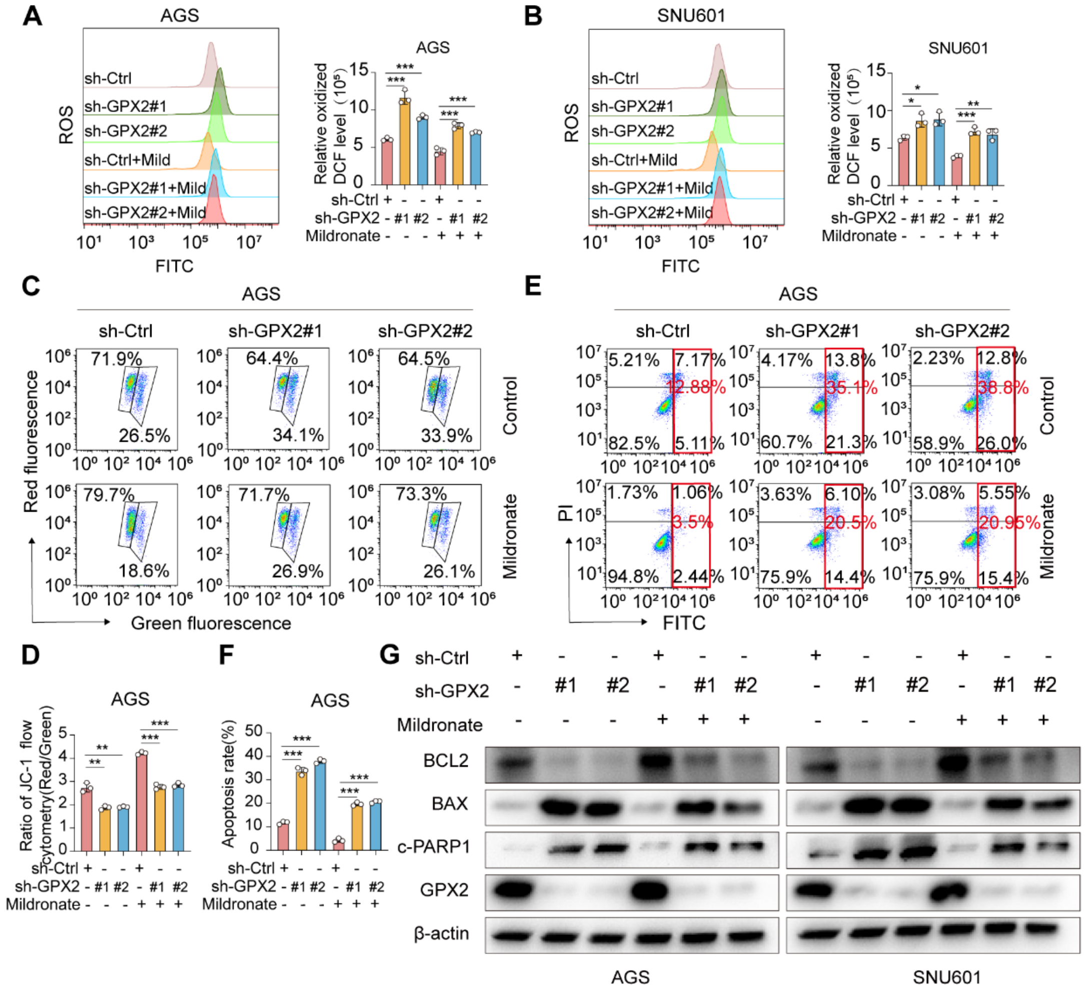

Supplementary Fig.11. Mildronate rescues GPX2 knockdown-induced mitochondrial dysfunction and apoptosis. (A-B) Flow cytometric analysis of ROS levels in GPX2-knockdown AGS and SNU601 cells treated with or without 5 mM mildronate for 24 h. (C-D) Flow cytometric analysis of mitochondrial membrane potential (JC-1 assay) in GPX2-knockdown AGS and SNU601 cells treated with or without 5 mM mildronate for 24 h. (E-F) Flow cytometric analysis of apoptosis in GPX2-knockdown AGS and SNU601 cells treated with or without 5 mM mildronate for 24 h. (G) Western blot analysis of apoptosis-related protein expression in GPX2-knockdown AGS and SNU601 cells treated with or without 5 mM mildronate for 24 h. The data are represented as the mean ±SD of three independent experiments. * $p < 0.05$; ** $p < 0.01$; *** $p < 0.001$, ns, no significance.

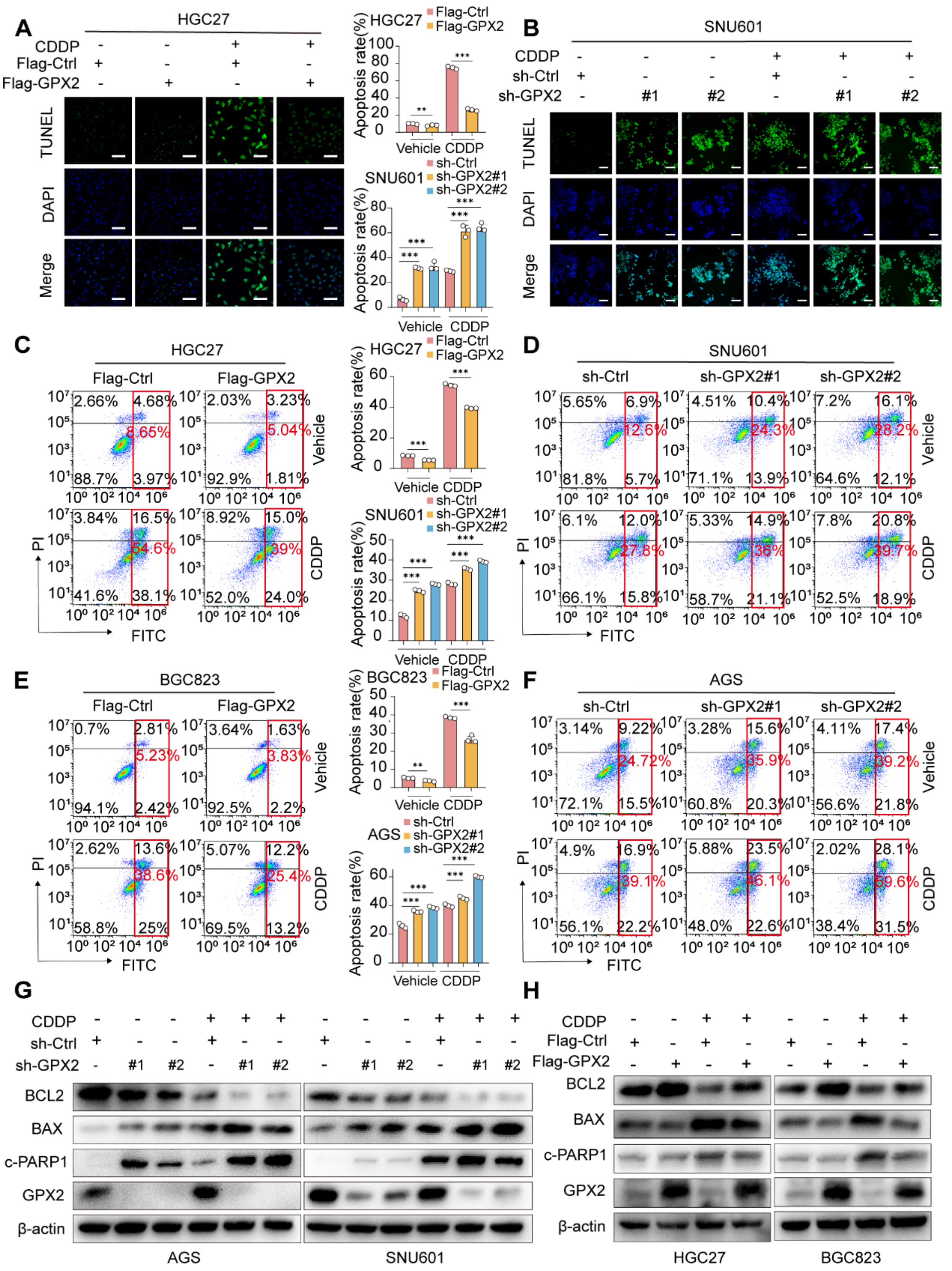

Supplementary Fig.12. Knockdown of GPX2 enhanced the sensitivity of gastric cancer cells to cisplatin-induced apoptosis. GPX2-overexpressed BGC823 cells (0.8 μg/ml), GPX2-overexpressed HGC27 cells (5 μg/ml), GPX2-knockdown AGS cells (1.6 μg/ml) and GPX2-knockdown SNU601 cells (2 μg / ml) were treated with cisplatin for 24 hours. (A)

and (B) The level of apoptosis was evaluated using the TUNEL assay. Scale: 100 μm. (C-F) Apoptotic rates were quantified using flow cytometry.(G-H) The expression of apoptosis-related proteins was analyzed by Western blot. The data are represented as the mean ±SD of three independent experiments. * $p < 0.05$; ** $p < 0.01$; *** $p < 0.001$, ns, no significance.
