# Supplementary figures and images for: Targeting GPX2 to disrupt lipid homeostasis and enhance cisplatin sensitivity in diffuse gastric cancer
Source: Cell Death Discov. 2025 Oct 27;11:491. doi: 10.1038/s41420-025-02771-8 (PMC12559244; doi:10.1038/s41420-025-02771-8)

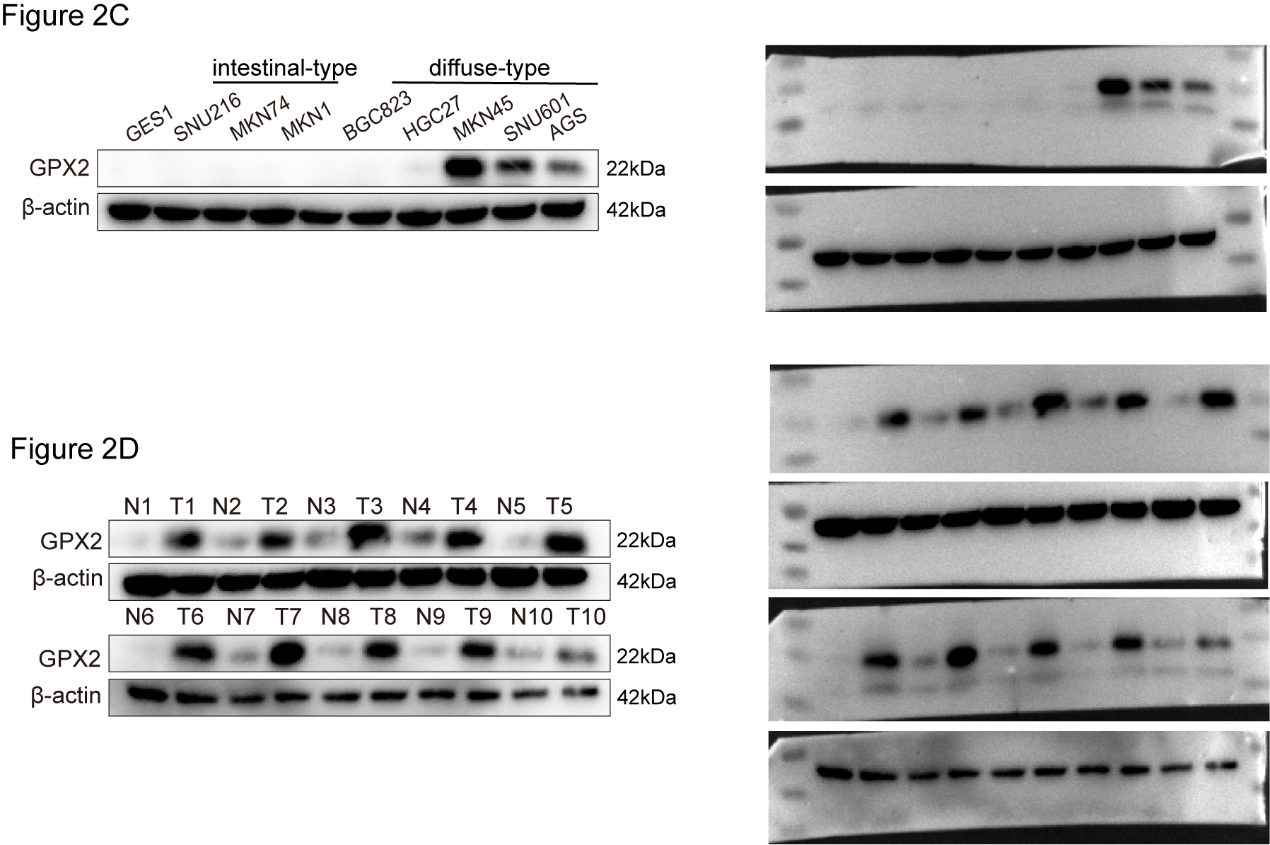


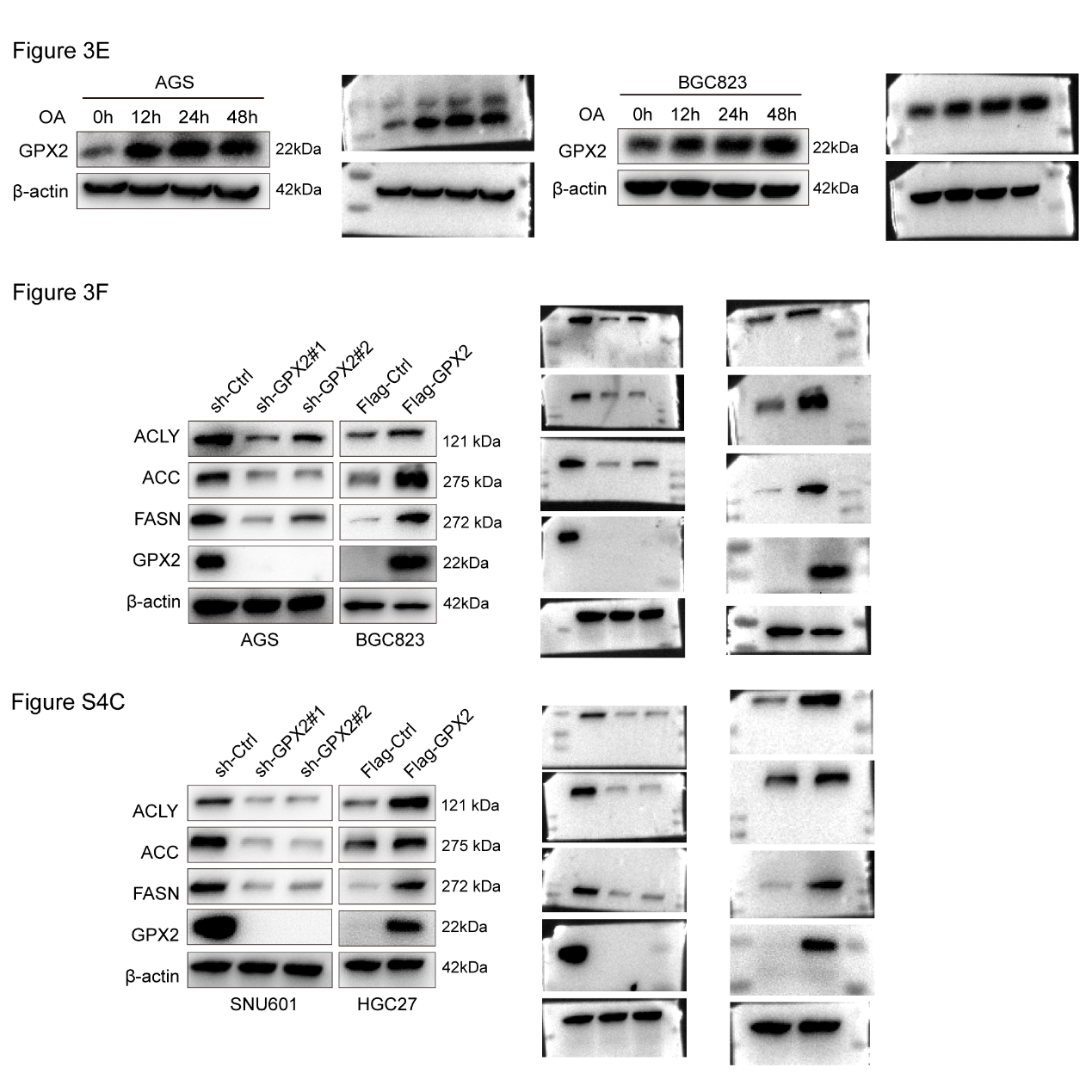


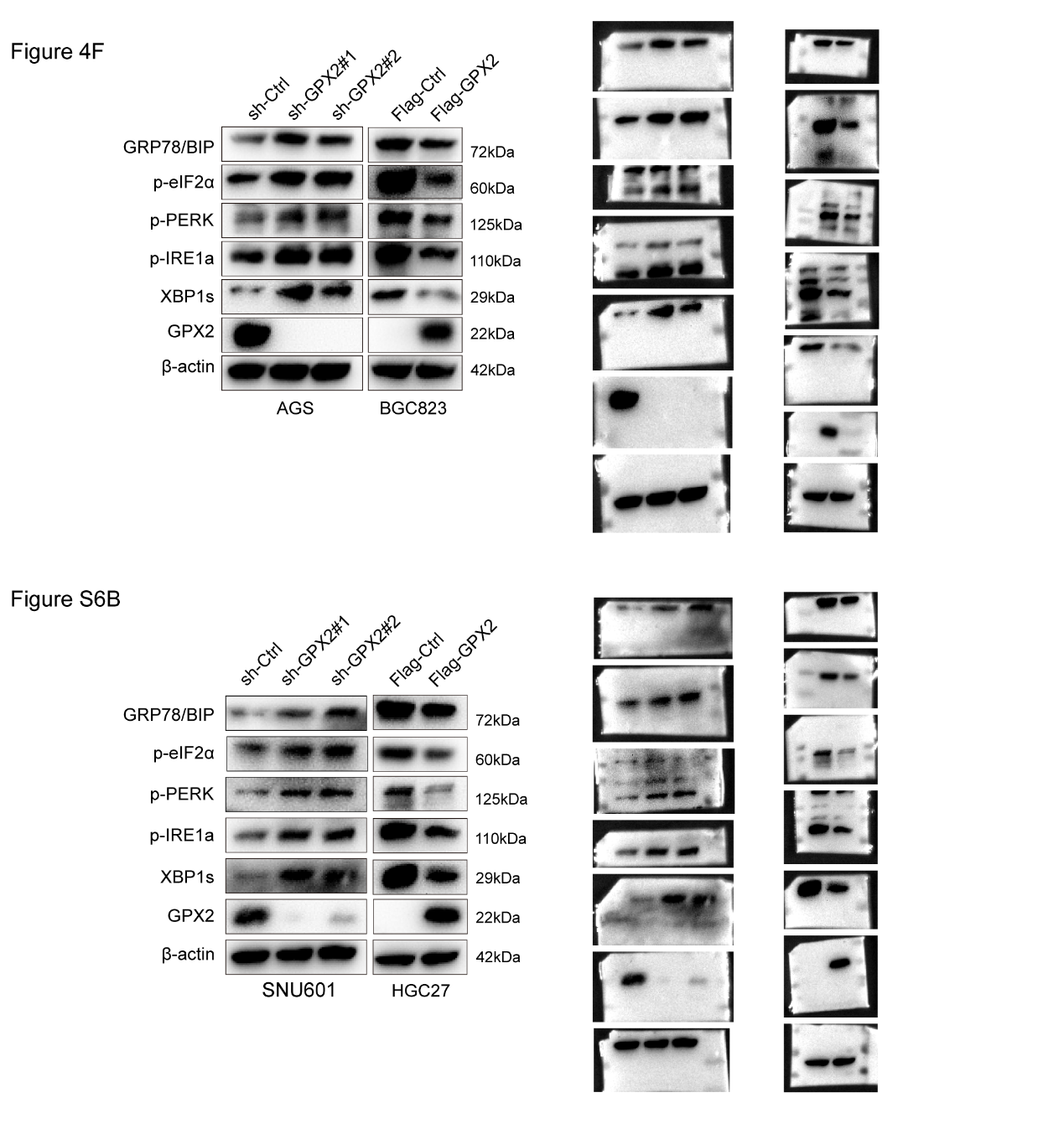


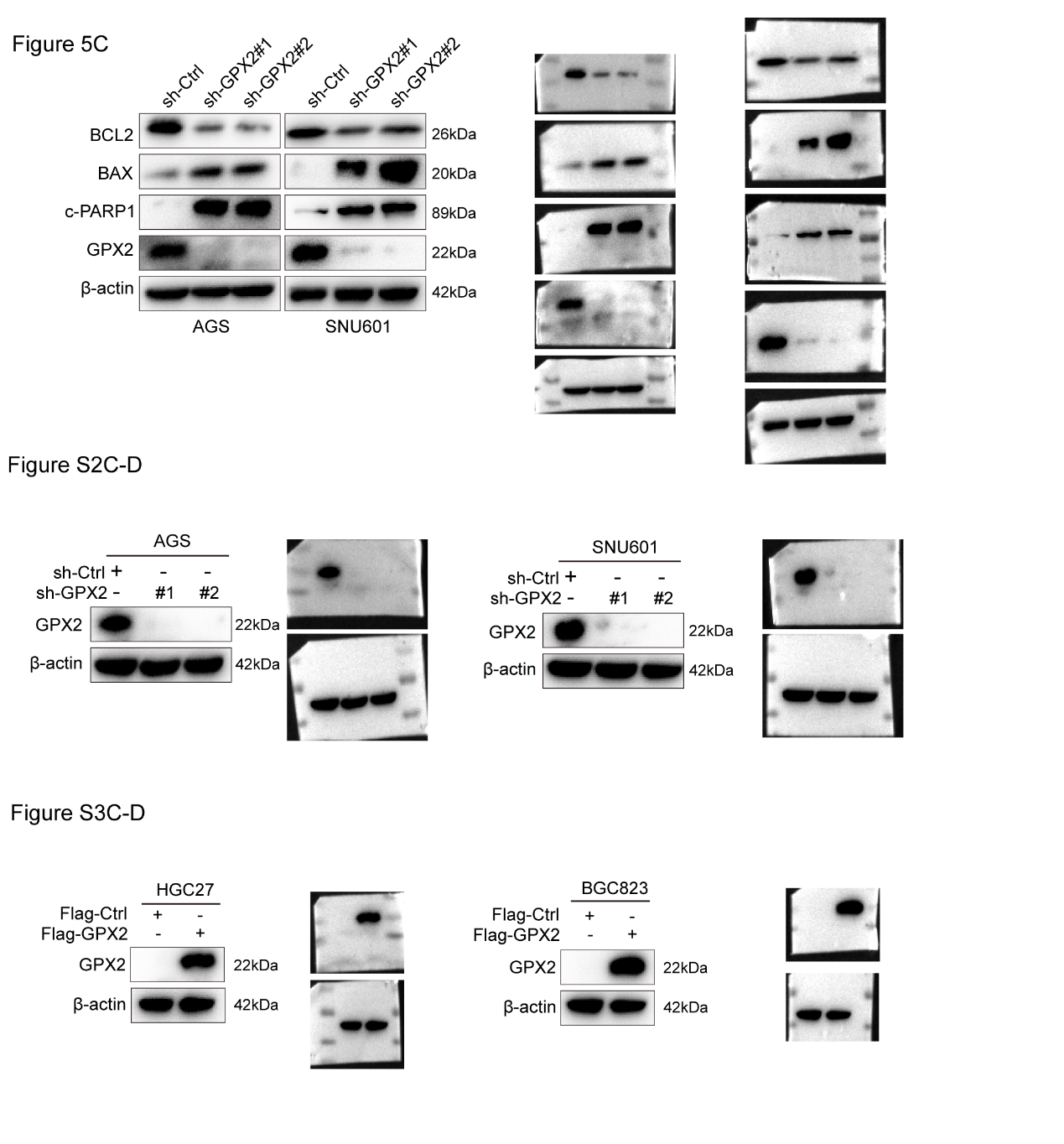


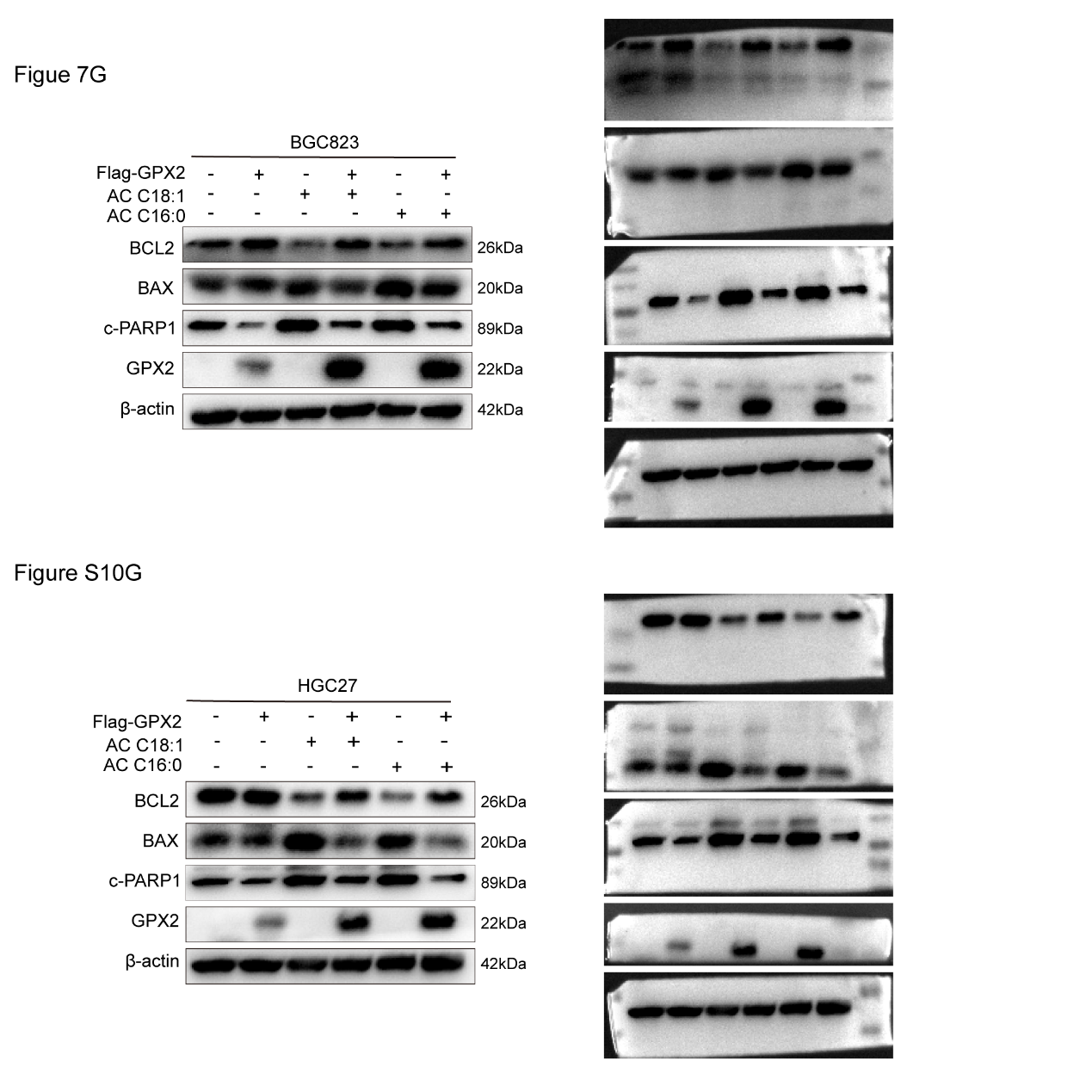


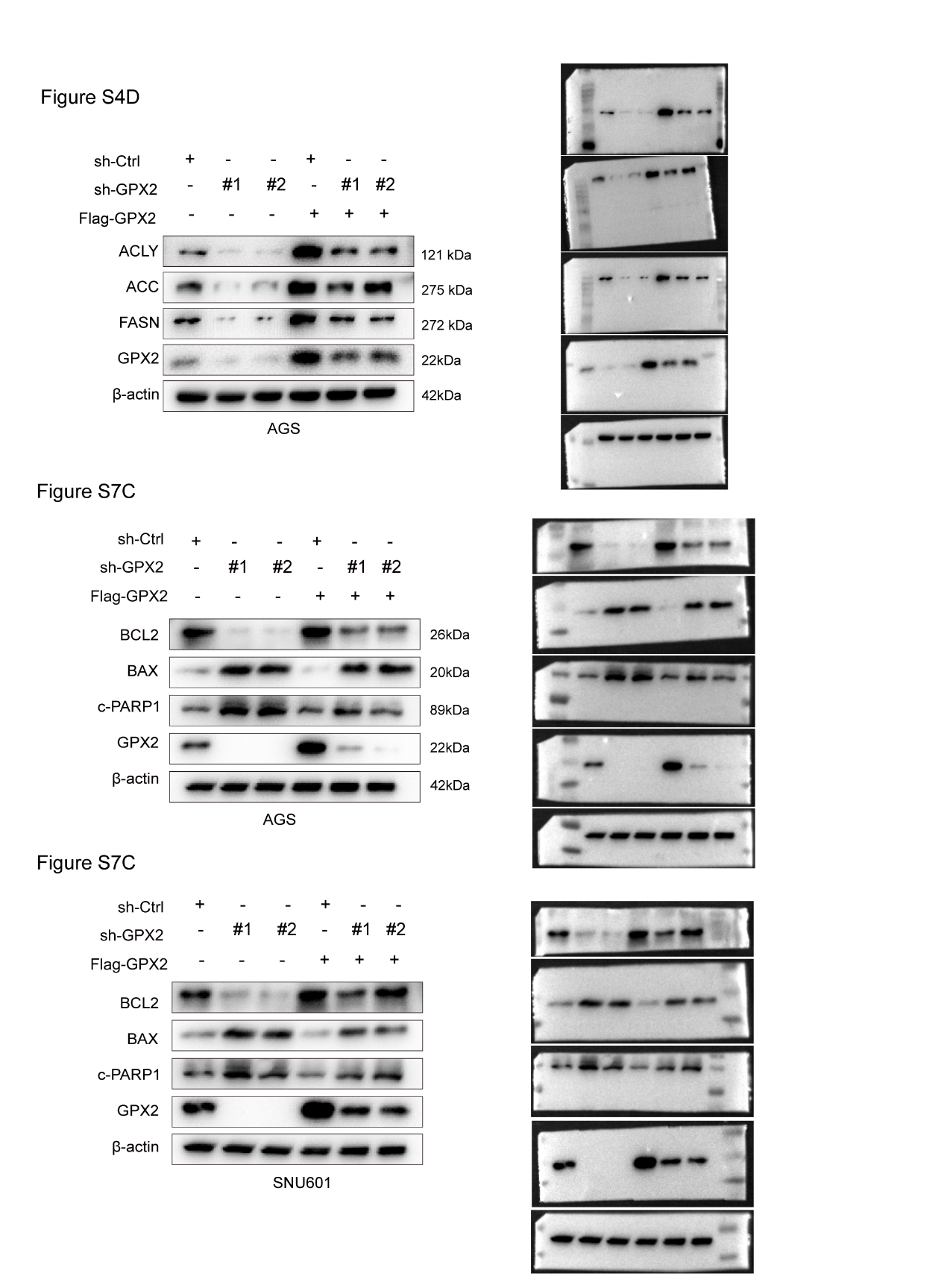


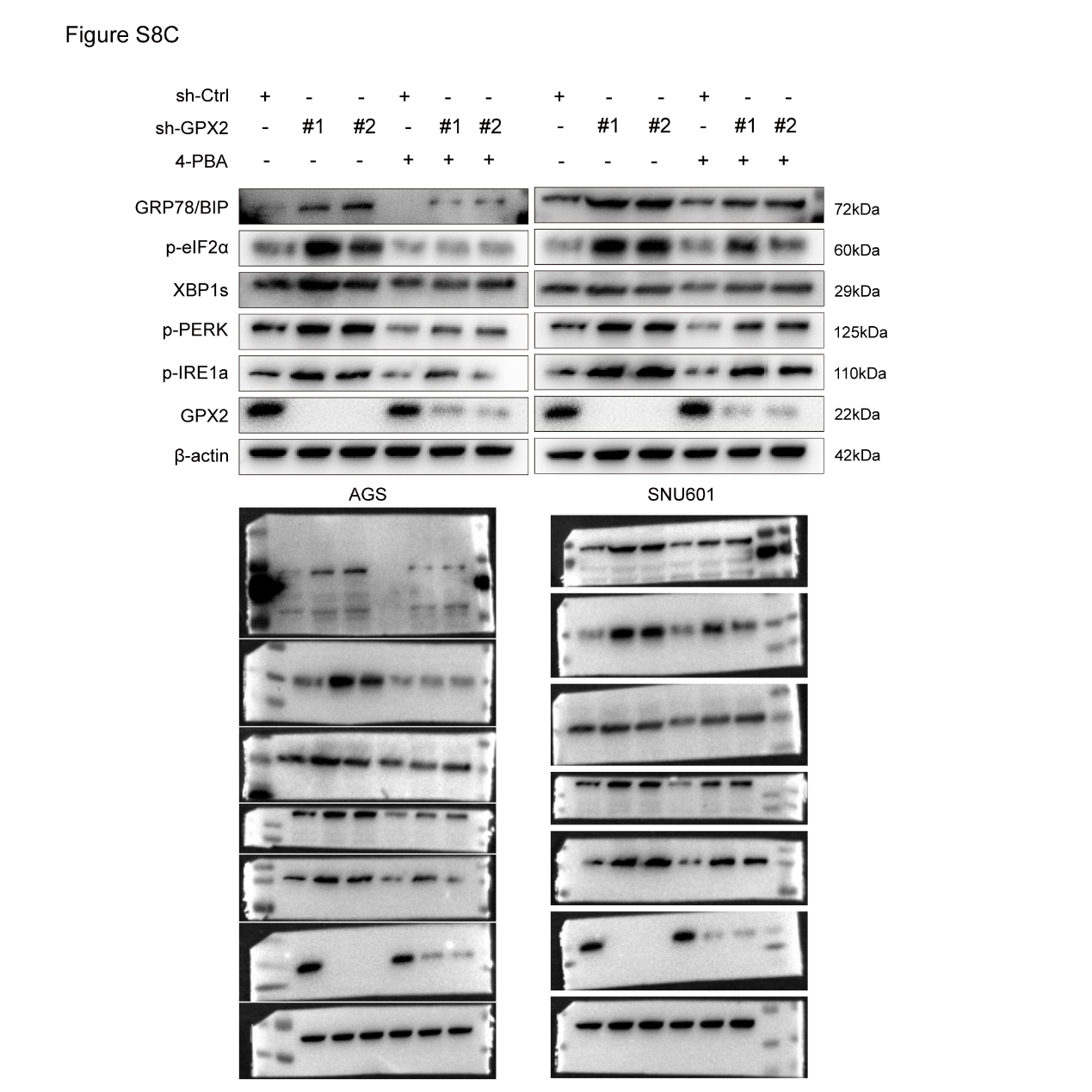


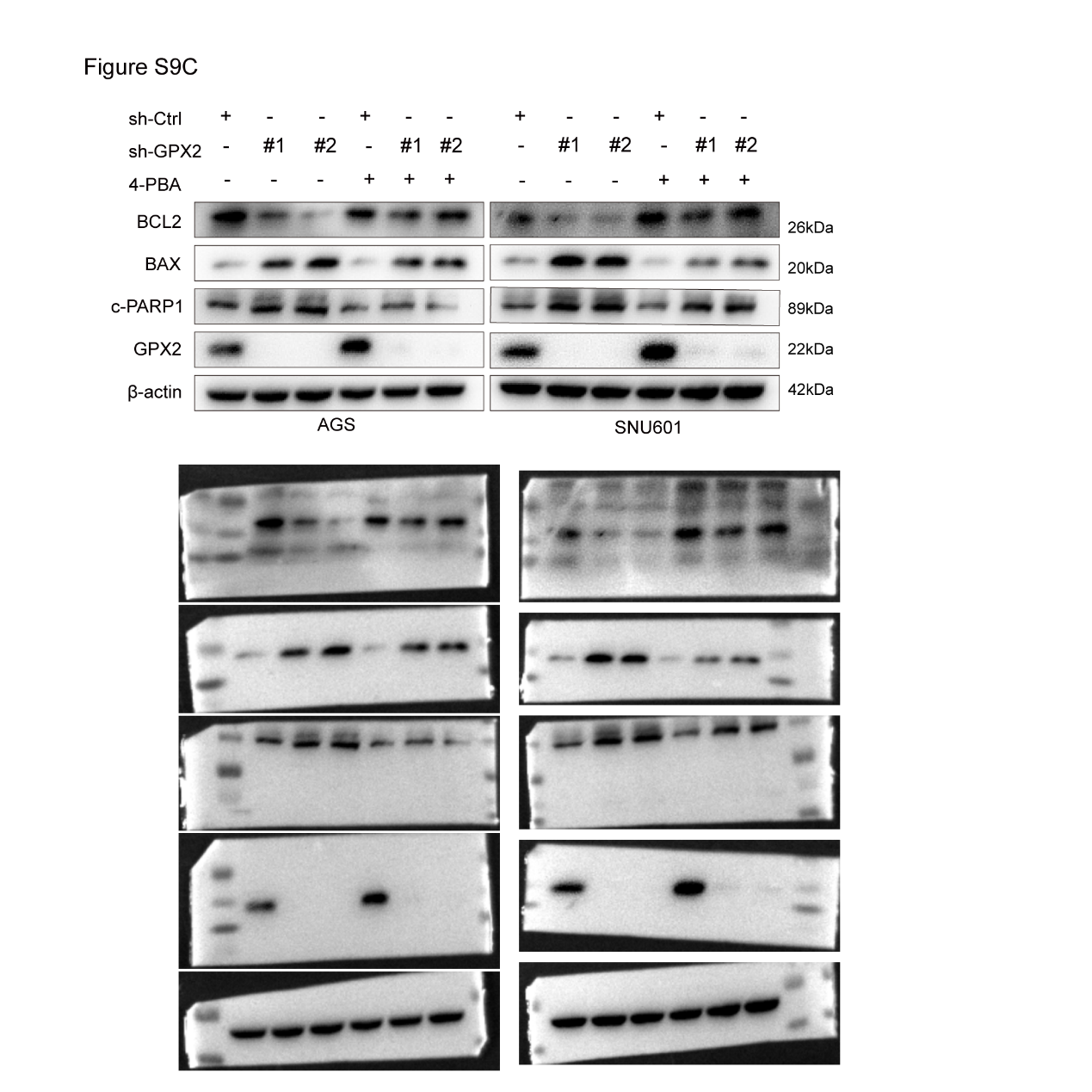


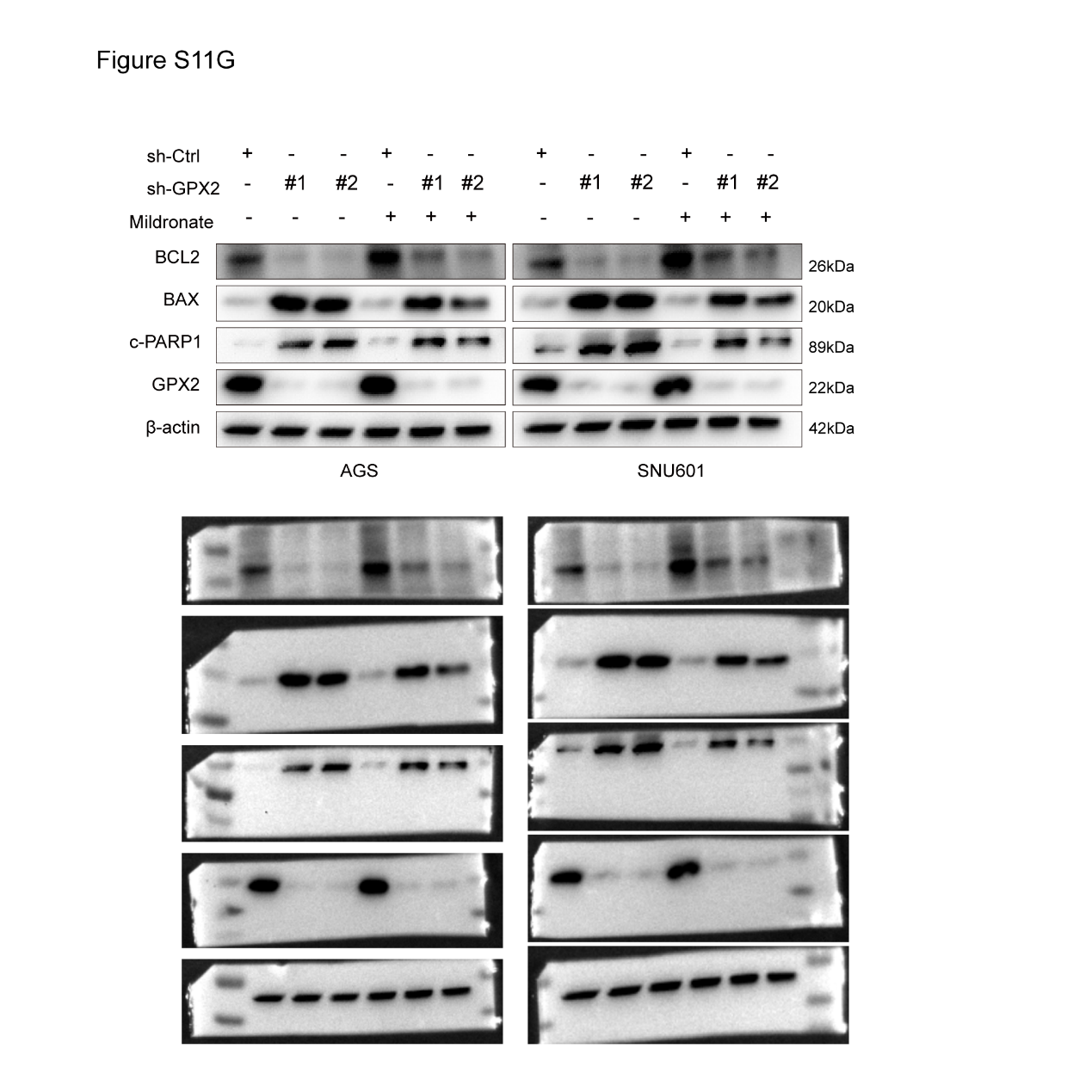


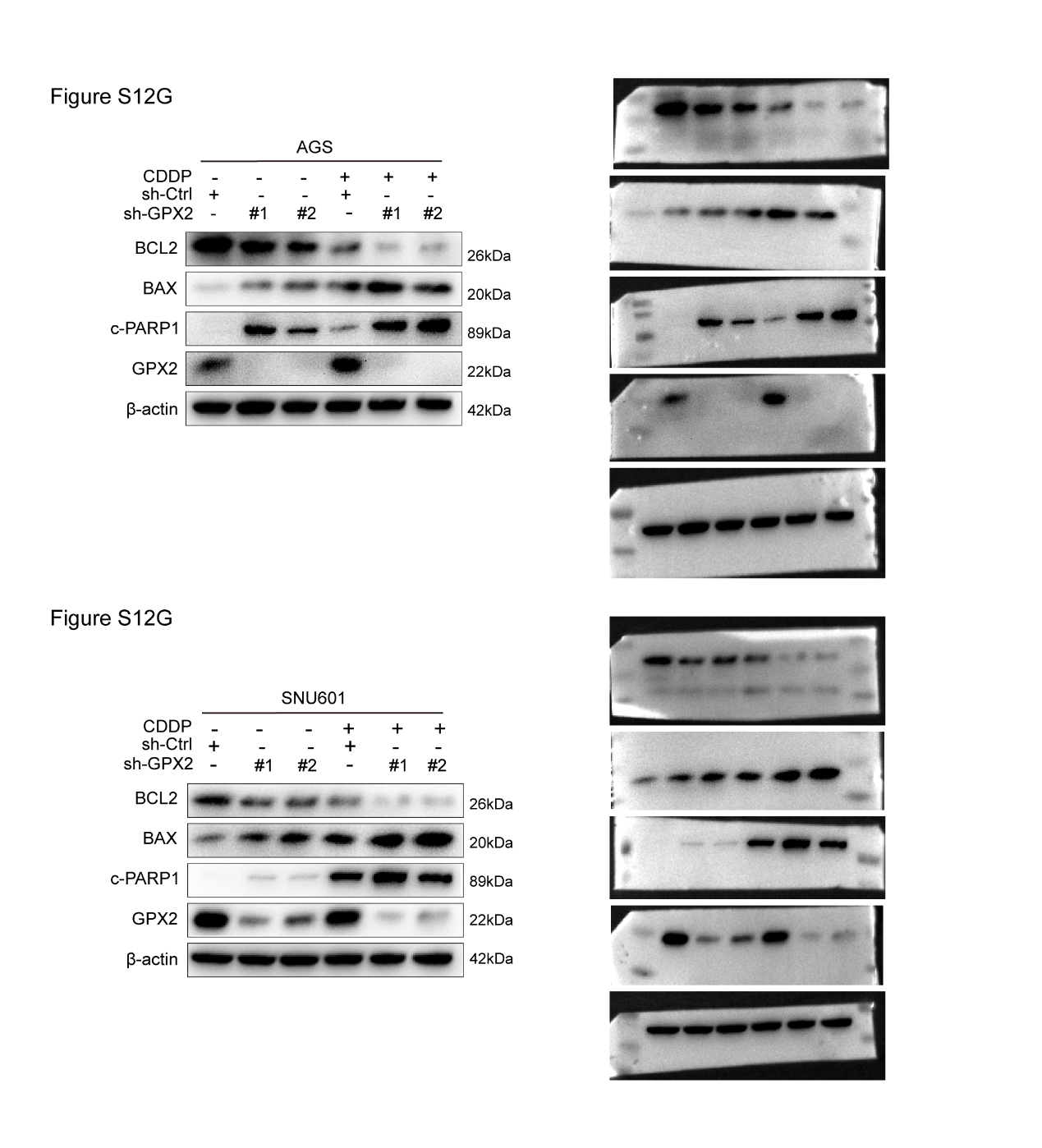

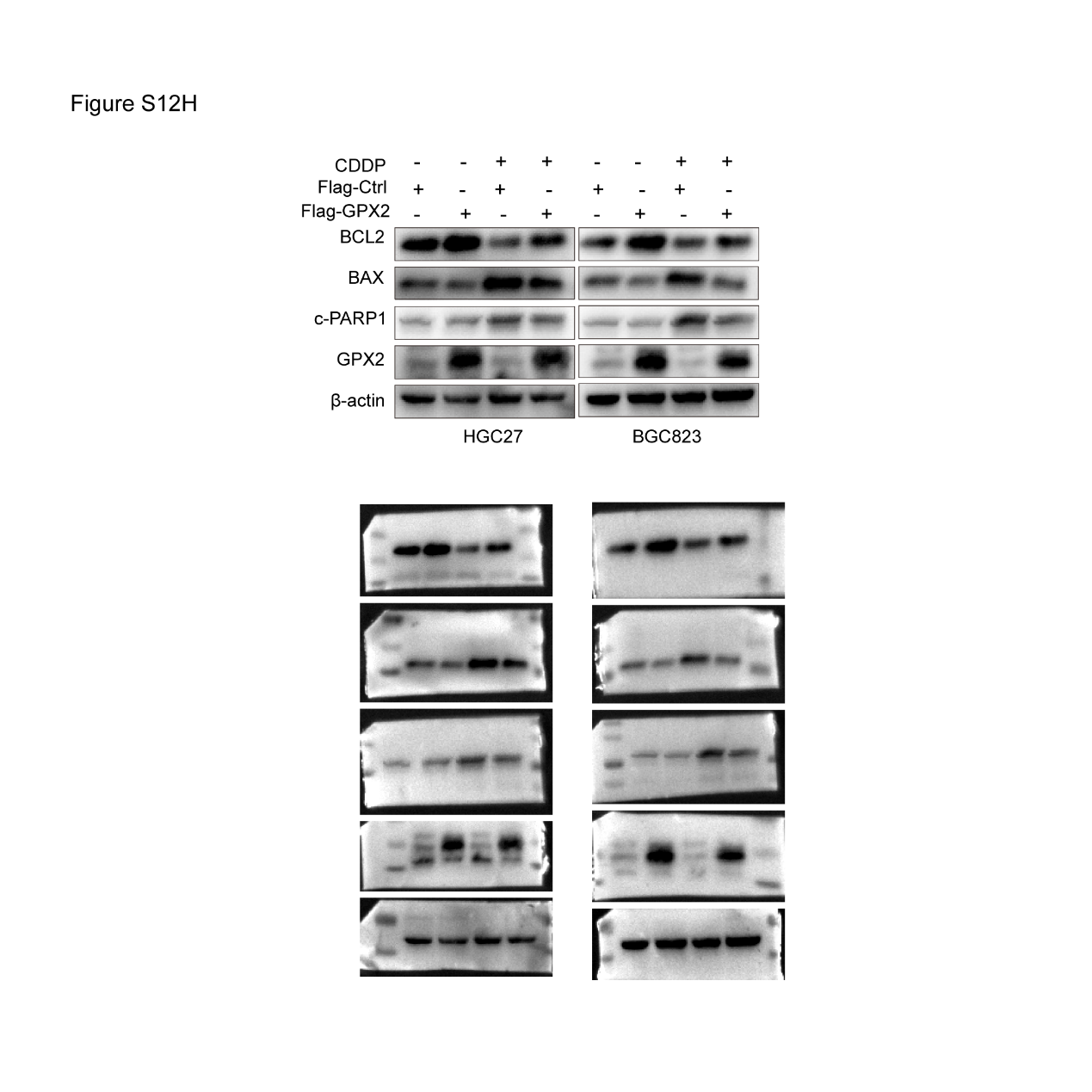

Supplement: Supplementary file 2 — Orignal data [file 41420_2025_2771_MOESM2_ESM.docx]
